# Seroprevalence of chikungunya virus infection among HIV-infected adults in French Caribbean Islands of Martinique and Guadeloupe in 2015: A cross-sectional study

**DOI:** 10.1371/journal.pntd.0009267

**Published:** 2021-04-09

**Authors:** Elodie Curlier, Laurence Fagour, Cécile Herrmann-Storck, Adrien Staelen, Ingrid Vingadassalom, Sébastien Breurec, Sylvie Abel, Sandrine Pierre-François, Janick Jean-Marie, Cédric Laouénan, Raymond Césaire, Bruno Hoen, André Cabié

**Affiliations:** 1 Department of Infectious Diseases, CHU de la Guadeloupe, Pointe-à-Pitre, France; 2 INSERM Centre d’Investigation Clinique Antilles-Guyane, Cayenne, France; 3 Department of Microbiology, CHU de la Martinique, Fort-de-France, France; 4 Department of Microbiology, CHU de la Guadeloupe, Pointe-à-Pitre, France; 5 Department of Infectious Diseases, CHU de la Martinique, Fort-de-France, France; 6 Université des Antilles, EA 4537, Fort de France, France; 7 INSERM, IAME, UMR 1137; Université Paris Diderot, Paris, France; DoD - AFHSB, UNITED STATES

## Abstract

**Background:**

In 2014, a first outbreak of chikungunya hit the Caribbean area where chikungunya virus (CHIKV) had never circulated before.

**Methodology/Principal findings:**

We conducted a cross-sectional study to measure the seroprevalence of CHIKV immediately after the end of the 2014 outbreak in HIV-infected people followed up in two clinical cohorts at the University hospitals of Guadeloupe and Martinique. Study patients were identified during the first months of 2015 and randomly selected to match the age and sex distribution of the general population in the two islands. They were invited to complete a survey that explored the symptoms consistent with chikungunya they could have developed during 2014 and to have a blood sample drawn for CHIKV serology.

The study population consisted of 377 patients (198 in Martinique and 179 in Guadeloupe, 178 men and 199 women), 182 of whom reported they had developed symptoms consistent with chikungunya. CHIKV serology was positive in 230 patients, which accounted for an overall seroprevalence rate of 61% [95%CI 56–66], with only 153 patients who reported symptoms consistent with chikungunya. Most frequent symptoms included arthralgia (94.1%), fever (73.2%), myalgia (53.6%), headache (45.8%), and skin rash (26.1%).

**Conclusions/Significance:**

This study showed that the seroprevalence of CHIKV infection was 61% after the 2014 outbreak, with one third of asymptomatic infections.

**Trial registration:**

ClinicalTrials.gov NCT 02553369.

## Author summary

After the termination of the first documented outbreak of chikungunya in the Caribbean in 2014, the seroprevalence rate of CHIKV infection in French West Indies was estimated at 61% [95% CI 56.0–65.8] in a population-matched cohort of HIV-infected patients. This high seroprevalence may explain the abrupt and persistent cessation CHIKV circulation in these territories. One third of participants had not developed clinical symptoms of chikungunya.

## Introduction

In December 2013, local transmission of chikungunya virus (CHIKV) was identified in the Caribbean for the first time. A large outbreak ensued, which hit the French Caribbean Islands of Guadeloupe (1,434 km^2^) and Martinique (1,128 km^2^) in a single wave in 2014. That year the population sizes of the two territories were 403,750 and 381,326 inhabitants, respectively. After the outbreak terminated in January 2015, it was estimated that approximately 308,000 people had developed clinically overt chikungunya, i.e. approximately 40% of the population [1]. However, an accurate estimation of the attack rate of this outbreak, based on laboratory-confirmed cases, is lacking. There is no evidence that CHIKV had circulated in the Caribbean for at least 200 years and the whole population was immunologically naïve to CHIKV before 2014 [2]. This provided an opportunity to estimate the outbreak attack rate by measuring the CHIKV seroprevalence rate in the immediate aftermath of the outbreak in a population sample reproducing the age and sex structure of the two island populations. We did so in a population-based cohort of HIV-infected patients, based on the assumption that the distribution of the living places of HIV-infected patients matched that of the general population in the two islands where most people live in urban and peri-urban economic areas. We also estimated the proportion of asymptomatic CHIKV infections.

## Methods

### Ethics statement

The study was approved by the Comité de Protection des Personnes Sud-Ouest et Outre Mer III (IRB 2014-A01504-43) and is registered with ClinicalTrials.gov (NCT 02553369) (see S1 Protocol). Both oral and written information explained the objectives and characteristics of the study to patients and an informed consent was signed by each participant. All patient personal data and information were de-identified.

### Study design

In the French Caribbean Islands of Guadeloupe and Martinique, people living with HIV (PLWHIV) are cared for in the Department of Infectious Diseases of the University hospital of each island. We conducted a cross-sectional study within the cohorts of PLWHIV and cared for in these two hospitals, which accounted for 997 and 950 patients, respectively, in January 2015. These cohorts of PLWHIV are registered with ClinicalTrials.gov (NCT02898987). Each cohort is maintained using the same electronic medical record system (Nadis). All collected data are subject to quality control, which is done once yearly, independently of this study [3].

Eligible for selection in this study were adults (> 18 years old) followed for HIV infection at the two investigators centers (Martinique and Guadeloupe, French West Indies) living in these territories. We excluded patients who had lived more than 6 months in a risk area for chikungunya virus infection prior to 2014, patients with a chronic rheumatism that had been documented prior to the chikungunya outbreak, patients who had received a blood transfusion during year 2013, and patients who had planned to live outside French West Indies during the follow up period.

Within each cohort, eligible patients were sorted by age (18–34, 35–44, 45–54, 55–65, and >65 years) and sex, and assigned one the 10 resulting groups. The study sample was built up by randomly selecting in each of the 10 groups the number of patients that was appropriate to reflect the age and sex distribution of each island population, based on the most recent national population census data (S1 Table). Subjects identified this way were invited to participate in the study at their subsequent routine HIV outpatient clinic visit, which took place between February and September 2015. During this visit, patients who accepted to participate were enrolled after they signed an informed consent. They were then invited to answer a questionnaire exploring whether they had developed clinical symptoms consistent with chikungunya at any time of 2014 and, if this was the case, the date and type of clinical manifestations. A 5-ml blood sample was drawn and tested for CHIKV-specific immunoglobulin G (IgG) using a commercially available assay (Euroimmun Chikungunya IgM/IgG ELISA, Luebeck, Germany).

Patients’ demographics and relevant information were extracted from the Nadis database.

A total of 362 subjects were to be enrolled in order to be able to estimate an expected 40% seroprevalence rate with a 95% confidence interval of ±5%. The number of eligible patients was increased by 10% in each age and sex stratum, to take into account the possibility for a subject to decline participating in the study.

Seroprevalence of CHIKV infection was defined by the proportion of positive CHIKV IgG among the number of subjects tested. The proportion of asymptomatic cases was the number of asymptomatic patients divided by the number of subjects that tested positive for CHIKV. CHIKV seroprevalence and proportion of asymptomatic cases were expressed as percentages with 95% confidence intervals. The 95% confidence intervals (CIs) were calculated using the exact (Clopper–Pearson) method. Continuous variables were summarized using median and interquartile range and compared using Mann-Whitney test. Categorical variables were summarized using numbers and percentages and compared using Fisher exact test. Statistical tests were performed with Stata software, version 13 (StataCorp LP, College Station, TX, USA).

## Results

A total of 388 patients were enrolled but the result of CHIKV serology could not be obtained for 11. Therefore, the study population consisted of 377 patients (198 in Martinique and 179 in Guadeloupe). Patients’ characteristics are presented in Table 1.

**Table 1:** Main characteristics of the 377 participants.

	CHIKV IgG positive (n = 230)	CHIKV IgG negative (n = 147)	All patients (n = 377)	p
Age (years), median [IQR]	51.5 [40–63]	47 [37–58]	50 [39–61]	0.02
Female gender, no (%)	128 (57.5)	71 (48.3)	199 (52.8)	0.17
Under antiretroviral therapy, no (%)	224 (97.4)	140 (95.2)	364 (96.6)	0.39
CD4 count (/mm^3^), median [IQR]	626 [480–791]	599 [390–834]	621[439–809]	0.28
HIV RNA < 50 copies/mL, no (%)	198 (86.5)	123 (84.3)	321 (85.6)	0.55
Reported acute chikungunya in 2014, no (%)	153 (66.5)	29 (19.7)	182 (48.3)	0.0001
Fever, no (% of symptomatic cases)	112 (73.2)	20 (69.0)	132 (72.5)	0.71
Arthralgia, no (% of symptomatic cases)	144 (94.1)	26 (89.7)	170 (93.4)	0.41
Myalgia, no (% of symptomatic cases)	82 (53.6)	17 (58.6)	99 (54.4)	0.43
Headache, no (% of symptomatic cases)	70 (45.8)	13 (44.8)	83 (45.6)	1.00
Skin rash, no (% of symptomatic cases)	40 (26.1)	8 (27.6)	48 (26.4)	0.68

CHIKV IgG: chikungunya virus specific immunoglobulin G antibodies

CHIKV serology was positive in 230 patients, which translates in a CHIKV seroprevalence rate of 61.0% [95% CI 55.9–66.0]. The CHIKV seroprevalence rates were almost identical in Guadeloupe and Martinique. Seroprevalence rates were not significantly different in men and women, while there was a trend towards lower seroprevalence in youngest adults and higher seroprevalence in the oldest (see Table 2).

**Table 2:** Seroprevalence of CHIKV in patients in Martinique and Guadeloupe according to territory, gender, and age (N = 377).

	Seroprevalence % (n/N)	Confidence Intervals 95%
Territory		
Guadeloupe	62.0 (111/179)	[54.5–69.1]
Martinique	60.1 (119/198)	[52.9–67.0]
Gender		
Male	57.3 (102/178)	[49.7–64.7]
Female	64.3 (128/199)	[57.2–70.0]
Age, years		
18–34	50.8 (33/65)	[38.1–63.4]
35–44	61.4 (43/70)	[49.0–72.8]
45–54	56.5 (52/92)	[45.8–66.8]
55–65	65.4 (53/81)	[54.0–75.7]
> 65	71.0 (49/69)	[58.8–81.3]

Among the 230 patients who were seropositive for CHIKV, 153 recalled having had symptoms consistent with chikungunya in 2014, while 77 did not, which accounts for a proportion of asymptomatic cases of 33.5% [95% CI 27.4–39.6]. Symptoms most frequently reported by patients who were seropositive for CHIKV included arthralgia (94.1%), fever (73.2%), myalgia (53.6%), headache (45.8%), and skin rash (26.1%) (S1 Data). We also compared the distribution of these symptoms by levels of CD4 lymphocyte counts (below versus above 200/mm^3^) and of HIV viral loads (below vs above 50 HIV-RNA copies/mL) in both seropositive and seronegative patients (S2 Table).

## Discussion

The two main findings of this study are that the estimation of CHIKV seroprevalence was approximately 60% in both islands and a third of cases were asymptomatic. Both figures are higher than previously reported. Regarding seroprevalence, our figures are higher than those observed in volunteer blood donors at the end of the outbreak in the same area (48% in Guadeloupe and 41% in Martinique) [4] and at the end of the outbreak in the Reunion island in 2005 (38.2%) [5–6] or in Mayotte islands (37,2%) [7] but are very close to those observed after the outbreak in Haiti (57.9%) [8]. It is highly likely that seroprevalence was underestimated in blood donors, probably because recruitment of donors was biased towards subjects less exposed to CHIKV. Lower seroprevalence rates observed in Reunion Island probably results from the fact that this territory is both larger and less uniformly populated than Guadeloupe and Martinique islands. At any rate, our results may not result from a lack of specificity of the assay we used, since the specificity of the Euroimmun assay was demonstrated to be 95% or more for IgG [9]. The relatively high rate of asymptomatic cases we observed in our study (33%, vs 20–25% in prior outbreaks [7]) may have resulted from a recall bias that could have led people who had presented with minor, paucisymptomatic forms of the disease to have forgotten these manifestations, although this hypothesis appears relatively unlikely as patients were interrogated soon after the end of the outbreak. Moreover, when considering that 19.7% of noninfected patients reported having presented with symptoms consistent with chikungunya, the actual rate of asymptomatic forms of the disease might even be higher than 33%.

A genuine strength of our study is that we did our best to minimize any selection bias by analyzing a study population that matched the age and sex distribution of the two island populations.

On the other hand, we acknowledge that our study has some limitations. As already mentioned, a recall bias may have led to underestimate the rate of symptomatic forms of chikungunya. Besides, since the most common symptoms of chikungunya are nonspecific and may be observed in other arboviral or flu-like diseases, some patients may have misreported such symptoms as chikungunya manifestations. Likewise, the fact that our study population was made of HIV-infected people could have introduced a bias. Although there is no data suggesting that HIV infection might alter host susceptibility to or clinical manifestations of CHIKV infection [10], one cannot exclude that exposure to CHIKV might have been different in PLWHIV as compared to the general island populations. On the one hand, the distribution of the living places of PLWHIV in the two islands does not differ from that of the general population. On the other hand, we acknowledge that socio-economic characteristics of PLWHIV differ from those of the general population in several aspects. For instance, the proportion of immigrants is higher in PLWHIV than in the general population (17.0% vs 2.4% and 38.2% vs 4.5% in Martinique and Guadeloupe, respectively) (Nadis data) [11]. Likewise, in Guadeloupe the proportions of persons that are professionally active and highly educated are lower in PLWHIV than in the general population (29.2% vs 40.0% and 11.2% vs 20.0%, respectively), while this is not true in Martinique (42.6% vs 41.9% and 19.8% vs 21.7%, respectively) (Nadis data) [11]. Whether these differences may have been detrimental to the representativeness of our study population is uncertain, especially as it was shown in both islands that all urban and rural districts have been equally affected by the chikungunya outbreak in 2014. As a result, it is unlikely that socioeconomic differences may have had an impact on seroprevalence estimates. The very close seroprevalence rates observed in Martinique (60.1%) and in Guadeloupe (62.0%) may be regarded as one more argument against a differential impact of socioeconomic factors on the spread of CHIKV infection within these territories.

The high seroprevalence rate that we consistently found in both Caribbean islands probably explains the abrupt and persistent cessation CHIKV circulation in these territories. It also provides useful information for modelling the risk of development of a new outbreak in these territories. Given the long-term persistence and protective efficacy of anti-CHIKV antibodies [12], at least one generation replacement and the re-introduction of CHIKV will condition the reemergence of chikungunya in these territories [13].

## Supporting information

S1 Checklist STROBE Checklist. (DOC)

S1 Table Distribution of island populations by age and gender (source: 2011 national population census data) vs breakdown of study population in each island. (DOCX)

S2 Table Symptoms according to chikungunya virus specific immunoglobulin G, lymphocytes CD4 count and plasma HIV RNA. (DOCX)

S1 Data Chikungunya seroprevalence study data, FWI, 2015. (XLSX)

S1 Protocol Chikungunya seroprevalence study protocol, FWI, 2015. (PDF)
