# Supplementary material for: Seroprevalence of chikungunya virus infection among HIV-infected adults in French Caribbean Islands of Martinique and Guadeloupe in 2015: A cross-sectional study
Source: PLoS Negl Trop Dis. 2021 Apr 9;15(4):e0009267. doi: 10.1371/journal.pntd.0009267 (PMC8059839; doi:10.1371/journal.pntd.0009267)
Supplement: S1 Checklist — (DOC) [file pntd.0009267.s001.doc]

STROBE Statement—Checklist of items that should be included in reports of ***cross-sectional studies***

|  | Item No | Recommendation |  |  |  |
| --- | --- | --- | --- | --- | --- |
| **Title and abstract** | 1 | (*a*) Indicate the study’s design with a commonly used term in the title or the abstract |  | Done (title and abstract) |  |
|  |  | (*b*) Provide in the abstract an informative and balanced summary of what was done and what was found |  | Done |  |
| Introduction | | |  |  |  |
| Background/rationale | 2 | Explain the scientific background and rationale for the investigation being reported |  | Done |  |
| Objectives | 3 | State specific objectives, including any prespecified hypotheses |  | Done |  |
| Methods | | |  |  |  |
| Study design | 4 | Present key elements of study design early in the paper |  | Done  Lines 84-85 |  |
| Setting | 5 | Describe the setting, locat, ions, and relevant dates, including periods of recruitment, exposure, follow-up, and data collection |  | Done  Lines 84-86, 100-109 |  |
| Participants | 6 | (*a*) Give the eligibility criteria, and the sources and methods of selection of participants |  | Done  Lines 89-102 |  |
| Variables | 7 | Clearly define all outcomes, exposures, predictors, potential confounders, and effect modifiers. Give diagnostic criteria, if applicable |  | Done  Lines 114-116 |  |
| Data sources/ measurement | 8* | For each variable of interest, give sources of data and details of methods of assessment (measurement). Describe comparability of assessment methods if there is more than one group |  | Done  Lines 103-109 |  |
| Bias | 9 | Describe any efforts to address potential sources of bias |  | Done  Line 96-100 |  |
| Study size | 10 | Explain how the study size was arrived at |  | Done  Lines 110-113 |  |
| Quantitative variables | 11 | Explain how quantitative variables were handled in the analyses. If applicable, describe which groupings were chosen and why |  | Done  Line 109 |  |
| Statistical methods | 12 | (*a*) Describe all statistical methods, including those used to control for confounding |  | Done  Lines 117-123 |  |
|  |  | (*b*) Describe any methods used to examine subgroups and interactions |  | Done in chapter results: lines 135-137 and in chapter discussion: lines 184-190 |  |
|  |  | (*c*) Explain how missing data were addressed |  | Done in chapter results: lines 126-127 |  |
|  |  | (*d*) If applicable, describe analytical methods taking account of sampling strategy |  | Not applicable |  |
|  |  | (*e*) Describe any sensitivity analyses |  | No sensitivity analyses performed |  |
| Results | | |  |  |  |
| Participants | 13* | (a) Report numbers of individuals at each stage of study—eg numbers potentially eligible, examined for eligibility, confirmed eligible, included in the study, completing follow-up, and analysed |  | Done  Lines 126-128 |  |
|  |  | (b) Give reasons for non-participation at each stage |  | Not applicable |  |
|  |  | (c) Consider use of a flow diagram |  | Not essential |  |
| Descriptive data | 14* | (a) Give characteristics of study participants (eg demographic, clinical, social) and information on exposures and potential confounders |  | Done  Lines 127-128 and Table 1  One cohort |  |
|  |  | (b) Indicate number of participants with missing data for each variable of interest |  | Done  Line 126 |  |
| Outcome data | 15* | Report numbers of outcome events or summary measures |  | Done  Lines 133-137 |  |
| Main results | 16 | (*a*) Give unadjusted estimates and, if applicable, confounder-adjusted estimates and their precision (eg, 95% confidence interval). Make clear which confounders were adjusted for and why they were included |  | Not applicable |  |
|  |  | (*b*) Report category boundaries when continuous variables were categorized |  | Done  Line 133 and table 2 |  |
|  |  | (*c*) If relevant, consider translating estimates of relative risk into absolute risk for a meaningful time period |  | Not applicable |  |
| Other analyses | 17 | Report other analyses done—eg analyses of subgroups and interactions, and sensitivity analyses |  | Not applicable |  |
| Discussion | | |  |  |  |
| Key results | 18 | Summarise key results with reference to study objectives |  | Done  Lines 151-152 |  |
| Limitations | 19 | Discuss limitations of the study, taking into account sources of potential bias or imprecision. Discuss both direction and magnitude of any potential bias |  | Done  Lines 173-196 |  |
| Interpretation | 20 | Give a cautious overall interpretation of results considering objectives, limitations, multiplicity of analyses, results from similar studies, and other relevant evidence |  | Done  Lines 153-172 |  |
| Generalisability | 21 | Discuss the generalisability (external validity) of the study results |  | Done  Lines 197-200 |  |
| Other information | | |  |  |  |
| Funding | 22 | Give the source of funding and the role of the funders for the present study and, if applicable, for the original study on which the present article is based |  | No specific funding |  |

*Give information separately for exposed and unexposed groups.

**Note:** An Explanation and Elaboration article discusses each checklist item and gives methodological background and published examples of transparent reporting. The STROBE checklist is best used in conjunction with this article (freely available on the Web sites of PLoS Medicine at http://www.plosmedicine.org/, Annals of Internal Medicine at http://www.annals.org/, and Epidemiology at http://www.epidem.com/). Information on the STROBE Initiative is available at www.strobe-statement.org.
