# Supplementary material for: Seroprevalence of chikungunya virus infection among HIV-infected adults in French Caribbean Islands of Martinique and Guadeloupe in 2015: A cross-sectional study
Source: PLoS Negl Trop Dis. 2021 Apr 9;15(4):e0009267. doi: 10.1371/journal.pntd.0009267 (PMC8059839; doi:10.1371/journal.pntd.0009267)
Supplement: S1 Table — (DOCX) [file pntd.0009267.s002.docx]

**S1 Table: Distribution of island populations by age and gender (source: 2011 national population census data) vs breakdown of study population in each island**

Table S1a: Distribution of population in Martinique by age and gender (source: 2011 national population census data)

| Age | Male | | Female | | Total | |
| --- | --- | --- | --- | --- | --- | --- |
| 18 to 34 years, no, % | 32 833 | 11% | 38 392 | 13% | 71 225 | 24% |
| 35 to 44 years, no, % | 24 378 | 8% | 32 036 | 11% | 56 414 | 19% |
| 45 to 54 years, no, % | 28 734 | 10% | 34 312 | 11% | 63 046 | 21% |
| 55 to 65 years, no, % | 21 811 | 7% | 25 788 | 9% | 47 599 | 16% |
| 65 years or more, no, % | 26 596 | 9% | 35 763 | 12% | 62 359 | 21% |
| Total | 134 352 | 45% | 166 291 | 55% | 300 643 | 100% |

Table S1b: Breakdown of the study population in Martinique by age and gender

| Age | Male | | Female | | Total | |
| --- | --- | --- | --- | --- | --- | --- |
| 18 to 34 years, no, % | 22 | 11% | 25 | 13% | 47 | 24% |
| 35 to 44 years, no, % | 16 | 8% | 21 | 11% | 37 | 19% |
| 45 to 54 years, no, % | 19 | 10% | 23 | 11% | 42 | 21% |
| 55 to 65 years, no, % | 14 | 7% | 17 | 9% | 32 | 16% |
| 65 years or more, no, % | 18 | 9% | 24 | 12% | 41 | 21% |
| Total | 89 | 45% | 110 | 55% | 199 | 100% |

Table S1c: Distribution of population in Guadeloupe by age and gender (source: 2011 national population census data)

| Age | Male | | Female | | Total | |
| --- | --- | --- | --- | --- | --- | --- |
| 18 to 34 years, no, % | 33 589 | 11% | 38 944 | 13% | 72 533 | 24% |
| 35 to 44 years, no, % | 25 932 | 9% | 34 366 | 11% | 60 298 | 20% |
| 45 to 54 years, no, % | 28 559 | 10% | 33 124 | 11% | 61 683 | 21% |
| 55 to 65 years, no, % | 21 660 | 7% | 25 528 | 9% | 47 188 | 16% |
| 65 years or more, no, % | 24 463 | 8% | 32 967 | 11% | 57 430 | 19% |
| Total | 134 203 | 45% | 164 929 | 55% | 299 132 | 100% |

Table S1d: Breakdown of the study population in Guadeloupe by age and gender

| Age | Male | | Female | | Total | |
| --- | --- | --- | --- | --- | --- | --- |
| 18 to 34 years, no, % | 22 | 11% | 26 | 13% | 48 | 24% |
| 35 to 44 years, no, % | 17 | 9% | 23 | 11% | 40 | 20% |
| 45 to 54 years, no, % | 19 | 10% | 22 | 11% | 41 | 21% |
| 55 to 65 years, no, % | 14 | 7% | 17 | 9% | 31 | 16% |
| 65 years or more, no, % | 16 | 8% | 22 | 11% | 38 | 19% |
| Total | 89 | 45% | 110 | 55% | 199 | 100% |
