# Supplementary material for: Seroprevalence of chikungunya virus infection among HIV-infected adults in French Caribbean Islands of Martinique and Guadeloupe in 2015: A cross-sectional study
Source: PLoS Negl Trop Dis. 2021 Apr 9;15(4):e0009267. doi: 10.1371/journal.pntd.0009267 (PMC8059839; doi:10.1371/journal.pntd.0009267)
Supplement: S2 Table — (DOCX) [file pntd.0009267.s003.docx]

S2 Table: Symptoms according to chikungunya virus specific immunoglobulin G, lymphocytes CD4 count and plasma HIV RNA

|  | **CHIKV IgG positive** | | | | **CHIKV IgG negative** | | | |
| --- | --- | --- | --- | --- | --- | --- | --- | --- |
|  | CD4/mm^3^ | | HIV RNA copies/ml | | CD4/mm^3^ | | HIV RNA copies/ml | |
|  | ≤ 200 | > 200 | < 50 | ≥ 50 | ≤ 200 | > 200 | < 50 | ≥ 50 |
| Fever | 4 (100) | 108 (72.5) | 101 (73.2) | 10 (71.4) | 1 (100) | 19 (67.9) | 16 (64) | 4 (100) |
| Arthralgia | 4 (100) | 140 (94.0) | 129 (93.5) | 14 (100) | 1 (100) | 25 (89.3) | 24 (96) | 2 (50) |
| Myalgia | 3 (75) | 79 (53.0) | 74 (53.6) | 8 (57.1) | 0 (0) | 17 (60.7) | 15 (60.0) | 2 (50) |
| Headache | 2 (50) | 68 (45.6) | 65 (47.1) | 5 (35.7) | 0 (0) | 13 (46.4) | 11 (44.0) | 2 (50) |
| Skin rash | 0 (0) | 40 (26.9) | 35 (25.4) | 4 (28.6) | 0 (0) | 8 (28.6) | 7 (28.0) | 1 (25.0) |
