# Supplementary material for: Seroprevalence of chikungunya virus infection among HIV-infected adults in French Caribbean Islands of Martinique and Guadeloupe in 2015: A cross-sectional study
Source: PLoS Negl Trop Dis. 2021 Apr 9;15(4):e0009267. doi: 10.1371/journal.pntd.0009267 (PMC8059839; doi:10.1371/journal.pntd.0009267)
Supplement: S1 Protocol — (PDF) [file pntd.0009267.s005.pdf]

<u>Titre de la recherche</u>

ESTIMATION DU TAUX DE SÉROPRÉVALENCE A LA FIN DE LA PREMIERE EPIDEMIE DE CHIKUNGUNYA AUX ANTILLES FRANÇAISES AU SEIN D'UN ECHANTILLON DE PATIENTS SUIVIS POUR UNE INFECTION PAR LE VIH.

<u>Acronyme de la Recherche</u>

ChikVIH

**Code promoteur : 14/B/18**<br>**Numéro ID RCB : 2014-A01504-43**

PROTOCOLE DE RECHERCHE BIOMEDICALE

Recherche biomédicale hors produits de santé

Version n°3.0 du 23/04/2015

A reçu un avis favorable initial du CPP Sud-Ouest Outre-Mer III le 29/11/2014 et une autorisation initiale de l'ANSM le 05/11/2014
A reçu un avis favorable du CPP SOOM III le 25/03/2015 (MS 1) et l'autorisation de l'ANSM le 12/03/2015 (MSA 1)
A reçu un avis favorable du CPP SOOM III le JJ/MM/AAAA (MS 2) et l'autorisation de l'ANSM le JJ/MM/AAAA (MSA 2)

Cette recherche a obtenu le financement du CHU de Martinique

Promoteur
CHU de Martinique-CS 90632,
97261 Fort-de-France cedex

<u>Centre de Méthodologie et de Gestion des données</u> :
Centre d'Investigation Clinique-Epidémiologie Clinique Antilles-Guyane (CIC1424 AG)

Investigateur coordonnateur
Pr André CABIE
Service de Maladies infectieuses et Tropicales
CHU de Martinique-CS 90632,
97261 Fort-de-France cedex

<u>Unité de vigilance de la recherche clinique</u> :
Unité de sécurité et de vigilance de la recherche clinique
Anne Gimbert (pharmacien), Francesco Salvo (médecin), Valérie Marty (ARC), Sandrine Vautrat (ARC)
Direction de la Recherche Clinique et de l'Innovation-CHU de Bordeaux
12 rue Dubernat
33404 Talence cedex
Tel : 05-57-82-08-34
Fax : 05-57-82-12-62

HISTORIQUE DES MISES A JOUR DU PROTOCOLE

VERSION	DATE	RAISON DE LA MISE A JOUR
2.0	22/02/2015	MS –MSA 1 :<br>Changement d'Investigateur coordonnateur<br>Changement d'IP (centre de Guadeloupe)<br>Allongement de la durée d'inclusion (6 mois)<br>Modification des critères de non inclusion<br>Modification de l'objectif principal<br>Modification concernant le déroulement de la recherche
3.0	23/04/2015	MS- MSA2<br>Suppression d'un critère d'inclusion<br>Allongement de la durée d'inclusion (8 mois)

PRINCIPAUX CORRESPONDANTS

Promoteur
CHU de Martinique
CS 90632, 97261 Fort-de-France cedex

Investigateur coordonnateur
Pr. André Cabié
Service de Maladies infectieuses et Tropicales
CHU de Martinique CS 90632,
97261 Fort-de-France cedex
andre.cabie@chu-fortdefrance.fr

Services participants
Pr. André Cabié
Service de Maladies infectieuses et Tropicales
CHU de Martinique CS 90632,
97261 Fort-de-France cedex
andre.cabie@chu-fortdefrance.fr

Pr. Raymond Césaire
Laboratoire de Virologie
CHU de Martinique CS 90632,
97261 Fort-de-France cedex
raymond.cesaire@chu-fortdefrance.fr

Pr. Bruno Hoen
Service de Maladies infectieuses et Tropicales
Dermatologie et Médecine Interne
CHU de la Guadeloupe - Hôpital Ricou
BP 465
97159 Pointe à Pitre, Cedex, France
bruno.hoen@chu-guadeloupe.fr

Centre de Méthodologie et de Gestion des données

CIC Antilles-Guyane
Coordonnateur : Pr. Mathieu Nacher
Site de Martinique :
CHU de Martinique, BP 632,
97261 Fort-de-France cedex
Site de Guadeloupe :
CHU de Pointe-à-Pitre, BP 465,
97159 Pointe-à-Pitre cedex

Méthodologiste et Biostatisticien
Pr. André Cabié
CIC Antilles-Guyane
andre.cabie@chu-fortdefrance.fr

Data manager
Benoît Tressière
Tél : 0590 89 15 03
benoit.tressiere@chu-guadeloupe.fr

Attachés de recherche clinique
Janick Jean-Marie
Tél : 0596 59 26 97
janick.jean-marie@chu-fortdefrance.fr

Isabelle Calmont
Tél : 0596 55 23 02
isabelle.calmont@chu-fortdefrance.fr

Technicienne d'étude clinique
CHU de Pointe à Pitre

Ingrid Vingadassalom
Tél : 0590 89 15 03
ingrid.vingadassalom@chu-guadeloupe.fr

PAGE DE SIGNATURE DU PROTOCOLE

Titre de la recherche

ESTIMATION DU TAUX DE SÉROPRÉVALENCE A LA FIN DE LA PREMIERE EPIDEMIE DE CHIKUNGUNYA AUX ANTILLES FRANÇAISES AU SEIN D'UN ECHANTILLON DE PATIENTS SUIVIS POUR UNE INFECTION PAR LE VIH.

Titre abrégé

ChikVIH

**Code promoteur : 14/B/18**
**Numéro ID RCB : 2014-A01504-43**
**Numéro d'enregistrement dans Clinicaltrials.gov :**

PROTOCOLE DE RECHERCHE BIOMEDICALE

Recherche biomédicale hors produits de santé

Version n°3.0 du 23/04/2015

A reçu un avis favorable initial du CPP Sud-Ouest Outre-Mer III le 29/11/2014 et une autorisation initiale de l'ANSM le 05/11/2014
A reçu un avis favorable du CPP SOOM III le 25/03/2015 (MS 1) et l'autorisation de l'ANSM le 12/03/2015 (MSA 1)
A reçu un avis favorable du CPP SOOM III le JJ/MM/AAAA (MS 2) et l'autorisation de l'ANSM le JJ/MM/AAAA (MSA 2)

Cette recherche a obtenu le financement du CHU de Martinique

Promoteur
CHU de Martinique - CS 90632,
97261 Fort-de-France cedex

A Fort de France le : 23/04/2015

M. Nicolas Estienne
Directeur Général du CHU de Martinique

Investigateur coordonnateur
Pr André CABIE
Service de Maladies infectieuses et Tropicales
CHU de Martinique CS 90632,
97261 Fort-de-France cedex

A Fort de France le :

Pr. André CABIE
Investigateur coordonnateur/PU-PH

PAGE DE SIGNATURE DU PROTOCOLE

Titre de la recherche

ESTIMATION DU TAUX DE SÉROPRÉVALENCE A LA FIN DE LA PREMIERE EPIDEMIE DE CHIKUNGUNYA AUX ANTILLES FRANÇAISES AU SEIN D'UN ECHANTILLON DE PATIENTS SUIVIS POUR UNE INFECTION PAR LE VIH.

Titre abrégé

ChikVIH

**Code promoteur : 14/B/18**
**Numéro ID RCB : 2014-A01504-43**

PROTOCOLE DE RECHERCHE BIOMEDICALE

Recherche biomédicale hors produits de santé

Version n°3.0 du 23/04/2015

A reçu un avis favorable initial du CPP Sud-Ouest Outre-Mer III le 29/11/2014 et une autorisation initiale de l'ANSM le 05/11/2014
A reçu un avis favorable du CPP SOOM III le 25/03/2015 (MS 1) et l'autorisation de l'ANSM le 12/03/2015 (MSA 1)
A reçu un avis favorable du CPP SOOM III le JJ/MM/AAAA (MS 2) et l'autorisation de l'ANSM le JJ/MM/AAAA (MSA 2)

Cette recherche a obtenu le financement du CHU de Martinique

Investigateur
Dr Elodie CURLIER
Service de Maladies infectieuses et Tropicales
Dermatologie et Médecine Interne
CHU de la Guadeloupe - Hôpital Ricou
BP 465
97159 Pointe à Pitre, Cedex, France

A Pointe à Pitre le :

Dr. Elodie CURLIER
Praticien hospitalier

Sommaire

1 RESUME DE LA RECHERCHE 9

2 CONTEXTE ET OBJECTIFS 13

2.1 CONTEXTE 13
2.2 OBJECTIFS 13
2.2.1 Objectif principal 13
2.2.2 Objectifs secondaires 13

3 METHODE 14

3.1 SCHEMA DE LA RECHERCHE 14
3.1.1 Mesures de séroprévalence 14
3.1.2 Détection des cas symptomatiques de chikungunya à la phase aigüe 14
3.1.3 Détection des formes chroniques de chikungunya 14
3.2 POPULATION DE L'ETUDE 14
3.2.1 Description de la population cible 14
3.2.2 Critères d'inclusion 15
3.2.3 Critères de non inclusion 15
3.2.4 Modalité de recrutement 15
3.2.5 Définition du moment d'inclusion dans l'enquête 15

4 DONNEES RECUEILLIES 15

4.1 DONNEES RECUEILLIES CONCERNANT L'INFECTION PAR LE CHIKUNGUNYA A LA PHASE AIGÜE 15
4.2 DONNEES RECUEILLIES CONCERNANT L'INFECTION PAR LE CHIKUNGUNYA A LA PHASE CHRONIQUE 16

5 CRITERES DE JUGEMENT 16

5.1 CRITERES DE JUGEMENT PRINCIPAL 16
5.2 CRITERES DE JUGEMENT SECONDAIRE 16

6 DEROULEMENT ET ORGANISATION DE L'ENQUETE 16

6.1 CALENDRIER DE LA RECHERCHE 16
6.2 RECRUTEMENT DES CENTRES INVESTIGATEURS 16
6.3 MODALITE D'INCLUSION ET RECUEIL DU CONSENTEMENT, DU QUESTIONNAIRE ET DES ECHANTILLONS BIOLOGIQUES 16
6.3.1 Tirage au sort 16
6.3.2 Rôle des Médecins investigateurs 17
6.4 SEROLOGIES 18
6.4.1 Organisation des analyses 18
6.4.2 Réalisation des analyses sérologiques 18
6.4.3 Résultats des analyses sérologiques 18
6.5 RENDU DES RESULTATS INDIVIDUELS 18

7 GESTION DES EVENEMENTS INDESIRABLES 19

7.1 DEFINITIONS 19
7.2 ROLE DE L'INVESTIGATEUR ET NOTIFICATION DES EVENEMENTS INDESIRABLES, GROSSESSE, FAIT NOUVEAU DE SECURITE 19
7.3 EVENEMENT INDESIRABLE GRAVE ATTENDUS 20
7.3.1 Période de notification 20
7.3.2 Rôle de l'unité de sécurité et de vigilance du CHU de Bordeaux 20
7.3.3 Rapport annuel de sécurité 21

8 ASPECTS STATISTIQUES 21

8.1 CONSTITUTION DE L'ECHANTILLON A ETUDIER 21
8.1.1 Calcul du nombre de sujets nécessaires 21

8.1.2 Répartition du nombre de sujets nécessaires 21
8.1.3 Répartition géographique des centres recruteurs 21
8.2 METHODES STATISTIQUES EMPLOYEES 21
9 SURVEILLANCE DE LA RECHERCHE 22
10 DROITS D'ACCES AUX DONNEES ET DOCUMENTS SOURCES 22
10.1 ACCES AUX DONNEES 22
10.2 DONNEES SOURCES 22
10.3 CONFIDENTIALITE DES DONNEES 22
11 CONTROLE ET ASSURANCE QUALITE 23
11.1 CONSIGNES POUR LE RECUEIL DES DONNEES 23
11.2 SUIVI DE LA RECHERCHE 23
11.3 CONTROLE DE QUALITE 23
12 CONSIDERATIONS ETHIQUES ET REGLEMENTAIRES 24
12.1 CADRE REGLEMENTAIRE DE L'ENQUETE 24
12.2 ENGAGEMENT DU PROMOTEUR ET DES INVESTIGATEURS 24
12.3 COMITE DE PROTECTION DES PERSONNES ET ANSM 24
12.4 AMENDEMENT AU PROTOCOLE 25
12.5 INFORMATION DU PATIENT ET FORMULAIRE DE CONSENTEMENT ECLAIRE ECRIT 25
12.6 ASSURANCES 25
12.7 TRAITEMENT INFORMATIQUE DES DONNEES 25
13 FINANCEMENT DE LA RECHERCHE 26
14 TRAITEMENT DES DONNEES ET CONSERVATION DES DOCUMENTS ET DES DONNEES RELATIVES A LA RECHERCHE 26
14.1 TRAITEMENT DES DONNEES 26
14.1 CONSERVATION DES DOCUMENTS RELATIFS A LA RECHERCHE 27
15 REGLES RELATIVES A LA PUBLICATION 27
15.1 COMMUNICATIONS SCIENTIFIQUES 27
15.2 COMMUNICATION DES RESULTATS AUX PATIENTS 27
15.3 CESSION DES DONNEES 27
16 REFERENCES BIBLIOGRAPHIQUES 28
17 ANNEXES 29
17.1 ANNEXE 1 : LISTE DES INVESTIGATEURS 29
17.2 ANNEXE 2 : COMPOSITION DU COMITE SCIENTIFIQUE DE L'ETUDE «CHIKVIH» 29
17.3 ANNEXE 3 : DONNEES DEMOGRAPHIQUES (RECENSEMENT INSEE 2011) ET ECHANTILLONNAGE 30
17.4 ANNEXE 4: AVIS DU CPP SUD OUEST ET OUTRE MER III & ANSM 51
17.6 ANNEXE 5 : ATTESTATION D'ASSURANCE 55
17.7 ANNEXE 6 : DECLARATION D'HELSINKI 56

LISTE DES ABREVIATIONS

ARC	Attaché de recherche clinique
ARH	Agence régionale de l'hospitalisation
ARS	Agence régionale de santé
CH	Centre hospitalier
CHU	Centre hospitalier universitaire
CIC	Centre d'investigation clinique – Epidémiologie clinique
CIRE	Cellule interrégionale d'épidémiologie
CNIL	Commission nationale informatique et liberté
CNR	Centre national de référence
CPP	Comité de protection des personnes
CRB	Centre de ressources biologiques
CRF	Case report form
DIRC	Délégation interrégionale à la recherche clinique
DSDS	Direction de la santé et du développement social
EA	Équipe d'accueil
ELISA	Enzyme-linked immunosorbent assay
ETP	Équivalent temps plein
IgG	Immunoglobulines de type G
INSEE	Institut national de la statistique et des études économiques
SMIT	Service de maladies infectieuses et tropicales
TEC	Technicien d'études cliniques
VIH	Virus de l'immunodéficience humaine

1 Résumé de la recherche

**PROMOTEUR**	CHU de Martinique<br>CS 90632, 97261 Fort-de-France cedex
**INVESTIGATEUR COORDONNATEUR**	Pr André Cabié<br>Service de maladies infectieuses et tropicales<br>CHU de Martinique,<br>CS 90632, 97261 Fort-de-France cedex<br>Tél : 0596 55 23 01<br>andre.cabie@chu-fortdefrance.fr
**TITRE**	Estimation du taux de séroprévalence à la fin de la première épidémie de Chikungunya aux Antilles françaises au sein d'un échantillon de patients suivis pour une infection par le VIH.
**TITRE ABREGE**	ChikVIH
**NUMERO D'IDENTIFICATION**	Code promoteur : 14/B/18<br>Numéro ID RCB : 2014-A01504-43
**VERSION DU PROTOCOLE**	Version n°3.0 du 23/04/2015
**JUSTIFICATION / CONTEXTE**	Fin 2013, les premiers cas autochtones de chikungunya ont été observés aux Antilles françaises. A la fin du mois de mai 2014, la plupart des iles de la caraïbe étaient concernés par l'épidémie. La présence de vecteurs compétents pendant les périodes estivales dans les zones tempérées d'Amérique du Nord et d'Europe rend probable la diffusion de cette infection à ces territoires.<br>Au cours de l'épidémie de grande ampleur qui a touché la Réunion en 2005/2006, le taux d'attaque a été de 38%. La période la plus active a duré 3 mois.<br>Dans ce contexte, la connaissance du taux d'attaque de l'épidémie qui touche les Antilles est un enjeu important pour la gestion des épidémies et les travaux de modélisation.<br>Comme le virus du chikungunya n'avait jamais circulé aux Antilles, la détermination du taux de séroconversion peut se faire en réalisant une enquête de séroprévalence en population générale à la fin de l'épidémie. Une autre méthode, plus simple, consiste à estimer ce taux au sein d'une cohorte de patients suivis régulièrement et dont l'habitat est réparti dans tout le territoire étudié. Le suivi des patients infectés par le virus de l'immunodéficience humaine (VIH) aux Antilles françaises se fait presque exclusivement en milieu hospitalier dans les services de maladies infectieuses et tropicales (SMIT). La forte prévalence du VIH et la répartition homogène des patients infectés sur l'ensemble de nos territoires, permettent de faire l'hypothèse que le risque de transmission des arbovirus par l'exposition aux piqûres de moustiques est comparable à celui de la population générale.<br>Ainsi ces patients qui bénéficient d'un suivi clinico biologique régulier, constituent un échantillon de population parfaitement adapté à l'étude de l'émergence du chikungunya aux Antilles françaises.
**OBJECTIFS**	**Objectif principal :**<br>Estimer le taux d'incidence cumulée à la fin de l'épidémie actuelle de chikungunya aux Antilles françaises par la mesure du taux de

	séroprévalence pour le chikungunya au sein d'un échantillon aléatoire de patients infectés par le VIH représentatif de la population générale de Martinique et de Guadeloupe.<br><br>**Objectifs secondaires :**<br>• Estimer la fréquence des infections asymptomatiques par le virus du chikungunya aux Antilles françaises dans la population étudiée.<br>• Décrire la répartition des infections symptomatiques par le virus du chikungunya aux Antilles françaises dans la population étudiée selon leur sévérité corrélée au type de prise en charge.<br>• Estimer la fréquence des formes chroniques de chikungunya dans la population étudiée.
**SCHEMA DE LA RECHERCHE**	Étude de cohorte avec mesures de séroprévalence.<br>Recherche biomédicale hors produits de santé, avec constitution d'une collection d'échantillons biologiques anonymisés (sans finalité de conservation).
**CRITERES D'INCLUSION**	• Patient(e) âgé(e) de 18 ans et plus<br>• Suivis pour leur infection par le VIH dans un des 2 centres participants<br>• Résidant aux Antilles françaises depuis au moins un an<br>• Affilié ou bénéficiaire d'un régime de sécurité sociale.<br>• Et ayant accepté de participer à l'étude, et signé(e) le consentement
**CRITERES DE NON-INCLUSION**	• Patient ayant séjourné plus de 6 mois dans une autre zone à risque de transmission du chikungunya<br>• Patient présentant ou ayant présenté un rhumatisme inflammatoire chronique<br>• Ayant bénéficié d'une transfusion de produits sanguins labiles durant l'année 2013 ou supposé en recevoir durant l'étude<br>• Ayant le projet de déménager hors des Antilles françaises durant la période de suivi
**DEROULEMENT DE LA RECHERCHE**	Les participants à la recherche seront recrutés dans le cadre du suivi de leur infection chronique par le VIH dans les centres participants.<br><br>• **Mesures de séroprévalence** avec recherche systématique des IgG spécifiques du chikungunya<br>***(1)*** ***Rétrospective :*** **à partir du plasma conservé au laboratoire de virologie de référence** (mesure de la charge virale VIH). Prélèvements réalisés dans le cadre du suivi biologique régulier de leur infection chronique par le VIH. Cette mesure initiale sera faite à partir de la plus récente mesure de charge virale effectuée en période pré épidémique. Elle a pour but de s'assurer de l'absence d'exposition antérieure au chikungunya<br>***(2)*** ***Prospective : Mesure finale*** à partir du plasma de la première mesure de la charge virale

	pratiquée dans les six mois suivant la fin de l'épidémie (date arrêtée par la CIRE Antilles Guyane). Reconvocation des patients hors du cadre du suivi habituel si nécessaire.<br><br>• **Détection des cas symptomatiques de chikungunya à la phase aigüe avec recueil de données cliniques évaluant la sévérité et le caractère atypique de l'épisode**<br>***(1) Rétrospective :*** le clinicien en charge du patient l'interrogera de façon orientée sur la survenue d'un épisode clinique évocateur de chikungunya depuis la dernière consultation.<br><br>• **Détection des formes chroniques de chikungunya**<br>Le clinicien référent en charge d'un patient séropositif pour le chikungunya lors de la mesure finale de séroprévalence, l'interrogera de façon orientée sur l'existence de symptômes cliniques évocateurs de formes chroniques d'infections par le chikungunya à l'occasion des visites de suivi de leur infection chronique par le VIH.<br>Cette détection des formes chroniques se fera de façon au moins semestrielle pendant les 18 mois qui suivront l'apparition des premiers symptômes évocateurs d'une infection par le virus du chikungunya.<br><br>• **Tests biologiques**<br>**Les IgG** spécifiques du chikungunya seront mesurées en ELISA.
**NOMBRE DE SUJETS NECESSAIRES**	362 patients sélectionnés après échantillonnage et répartis de façon proportionnelle sur les 2 centres participants (181 en Martinique et 181 en Guadeloupe). Effectifs par classe d'âge et par territoire établis à partir des données démographiques en population générale issues du recensement de l'INSEE de 2011. Effectif global calculé sur la base d'une séroprévalence équivalent au taux d'attaque estimé lors de l'épidémie survenue à la Réunion entre 2005 et 2006 (soit une prévalence de 38% pour un intervalle de confiance de 95%).
**NOMBRE PREVU DE CENTRES**	2 centres (CHU de Martinique - CHU de Pointe-à-Pitre)
**DUREE DE LA RECHERCHE**	Date théorique de début des inclusions: 01/01/2015<br>Durée de la période d'inclusion : 8 mois<br>Fin théorique de la période d'inclusion : 31/08/2015<br>Date de fin estimée de la recherche : 18 mois suivants le début des symptômes de chikungunya du dernier patient symptomatique inclus.
**ANALYSE STATISTIQUE DES DONNEES**	Analyse descriptive.<br>L'estimation du d'incidence cumulée se fera avec un intervalle de confiance bilatéral à 95%.
**RETOMBEES ATTENDUES**	• L'estimation du taux d'incidence cumulée de la première épidémie de chikungunya survenue aux Antilles françaises est une donnée essentielle pour les instances de santé publique pour évaluer a posteriori son impact sur le

	système de santé et anticiper son émergence sur d'autres territoires dans les Amériques.<br>• L'estimation de la proportion des formes asymptomatiques de chikungunya est variable selon les études. Son estimation est essentielle pour comprendre la dynamique de l'épidémie et son rôle dans l'immunité de groupe.<br>• L'estimation de la proportion des formes chroniques est essentielle pour évaluer la charge que leur prise en charge pourra représenter pour les systèmes de santé concernés.

2 Contexte et Objectifs

2.1 Contexte

Le chikungunya est un alphavirus de la famille des *Togaviridae*. Il s'agit d'une arbovirose transmise par la piqûre d'un moustique du genre Aedes (principalement *Aedes aegypti* et *Aedes albopictus*). C'est un vecteur diurne avec un pic d'activité en début et en fin de journée.

La présentation habituelle de la maladie comporte de la fièvre et des arthralgies invalidantes, qui parfois persistent des semaines, des mois ou même des années après l'épisode infectieux aiguë (1). Certaines présentations considérées comme atypiques ou compliquées ont été décrites comme des myocardites, des hépatites ou des méningo encéphalites (2). La proportion significative d'infections asymptomatiques fait débat avec des estimations qui varient de 3 à 28% selon les études (3–6). Une transmission sanguine est possible (6,7) ainsi qu'une transmission néonatale chez des mères virémiques dans les jours qui précédent l'accouchement (8,9).

Fin 2013, les premiers cas autochtones de chikungunya ont été observés aux Antilles françaises (10). A la fin du mois de mai 2014, la plupart des iles de la caraïbes étaient concernées par l'épidémie. La présence de vecteurs compétents pendant les périodes estivales dans les zones tempérées d'Amérique du Nord et d'Europe rend probable la diffusion de cette infection à ces territoires. Au cours de l'épidémie de grande ampleur qui a touché la Réunion en 2005/2006, le taux de séroconversion a été estimé à plus de 38% (11).

Dans ce contexte, la connaissance du taux de séroconversion de l'épidémie qui touche les Antilles est un enjeu important pour la gestion des épidémies à venir et les travaux de modélisation.

Etant donné que le virus du chikungunya n'avait encore jamais circulé aux Antilles (12), l'estimation du taux d'incidence cumulée peut se faire en réalisant une enquête de séroprévalence en population générale à la fin de l'épidémie ou plus simplement au sein d'une cohorte de patients suivis régulièrement et dont l'habitat est réparti dans tout le territoire étudié.
Le suivi des patients infectés par le virus de l'immunodéficience humaine (VIH) aux Antilles françaises se fait presque exclusivement en milieu hospitalier dans les services de maladies infectieuses et tropicales (SMIT). La forte prévalence du VIH et la répartition homogène des patients infectés sur l'ensemble de nos îles, permettent de faire l'hypothèse que le risque de transmission des arbovirus par l'exposition aux piqûres de moustiques est comparable à celui de la population générale.

Ainsi ces patients qui bénéficient d'un suivi clinico biologique régulier, constituent un échantillon de population parfaitement adapté à l'étude de l'émergence du chikungunya aux Antilles françaises.

2.2 Objectifs

2.2.1 Objectif principal

Estimer le taux d'incidence cumulée à la fin de l'épidémie actuelle de chikungunya aux Antilles françaises par la mesure du taux de séroprévalence pour le chikungunya au sein d'un échantillon aléatoire de patients infectés par le VIH représentatif de la population générale de Martinique et de Guadeloupe.

2.2.2 Objectifs secondaires

- Estimer la fréquence des infections asymptomatiques par le virus du chikungunya aux Antilles françaises dans la population étudiée.
- Décrire la répartition des infections symptomatiques par le virus du chikungunya aux Antilles françaises dans la population étudiée selon leur sévérité corrélé au type de prise en charge.

- Estimer la fréquence des formes chroniques de chikungunya dans la population étudiée.

3 Méthode

3.1 Schéma de la recherche

Les participants à la recherche seront recrutés dans le cadre du suivi de leur infection chronique par le VIH dans les centres participants.

3.1.1 Mesures de séroprévalence

Mesures de séroprévalence répétées par dosage des IgG spécifiques du chikungunya en ELISA.

1. ***Rétrospective :*** **à partir du plasma conservé au laboratoire de virologie de référence** (mesure de la charge virale VIH). Prélèvements réalisés dans le cadre du suivi biologique régulier de leur infection chronique par le VIH. Lorsqu'elle est possible cette mesure initiale se fera à partir de la plus récente mesure de charge virale effectuée en période pré épidémique. Elle a pour but de s'assurer de l'absence d'exposition antérieure au chikungunya.
2. ***Prospective :*** *mesure finale* à partir du plasma de la première mesure de la charge virale pratiquée dans les six mois suivant la fin de l'épidémie (date arrêtée par la CIRE Antilles Guyane). Reconvocation des patients hors du cadre du suivi habituel si nécessaire.

3.1.2 Détection des cas symptomatiques de chikungunya à la phase aigüe

Détection des cas symptomatiques de chikungunya à la phase aigüe avec recueil de données cliniques évaluant la sévérité et le caractère atypique de l'épisode

1. ***Rétrospective :*** le clinicien en charge du patient l'interrogera de façon orientée sur la survenue d'un épisode clinique évocateur de chikungunya depuis la dernière consultation.

3.1.3 Détection des formes chroniques de chikungunya

Elle se basera sur la survenue d'un des quatre principaux tableaux décrits dans la littérature (polyarthrite rhumatoïde, rhumatisme psoriasique avec ou sans atteinte axiale, périostites des poignets et des chevilles et douleurs/gonflements articulaires non spécifiques) à plus de trois mois d'un tableau évocateur d'un épisode aigue d'infection par le chikungunya, comme indiqué dans les recommandations nationales sur la prise en charge du chikungunya (cf annexes 5).

Le clinicien référent en charge d'un patient séropositif pour le chikungunya lors de la mesure finale de séroprévalence, l'interrogera de façon orientée sur l'existence de symptômes évocateurs de formes chroniques d'infections par le chikungunya et l'examinera de façon exhaustive sur le plan rhumatologique.

Ce dépistage des formes chroniques rentre dans le cadre des soins courants (visite de suivi systématique) et des bonnes pratiques médicales (avis spécialisé). Il sera donc réalisé de façon au moins semestrielle pendant les 18 mois suivants le début des symptômes de chikungunya du dernier patient symptomatique inclus.

3.2 Population de l'étude

3.2.1 Description de la population cible

Population cible constituée après échantillonnage représentatif de la population générale à partir des files actives de patients suivis pour leur infection chronique par le VIH dans les centres participants.

Effectifs répartis par territoire, sexe et classe d'âge selon les données du recensement démographique réalisé par l'INSEE en 2011.

3.2.2 Critères d'inclusion

- Patient(e) âgé(e) de 18 ans et plus
- Suivis pour leur infection par le VIH dans un des 4 centres participants
- Résidant aux Antilles françaises depuis au moins un an
- Affilié ou bénéficiaire d'un régime de sécurité sociale.
- Et ayant accepté de participer à l'étude et signé(e) le consentement

3.2.3 Critères de non inclusion

- Patient ayant séjourné plus de 6 mois dans une autre zone à risque de transmission du chikungunya
- Patient présentant ou ayant présenté un rhumatisme inflammatoire chronique
- Ayant bénéficié d'une transfusion de produits sanguins labiles durant l'année 2013 ou supposé en recevoir durant l'étude
- Ayant le projet de déménager hors des Antilles françaises durant la période de suivi

3.2.4 Modalité de recrutement

Le recrutement des patients satisfaisant les critères de sélection, se fera sur la base de leurs caractéristiques démographiques (sexe et classe d'âge). Les effectifs à atteindre par centre participant et répartis par sexe et par classe d'âge, seront transmis aux investigateurs avant la date de début des inclusions.

3.2.5 Définition du moment d'inclusion dans l'enquête

Les participants à cette étude seront recrutés par leur médecin spécialiste réferent sur une période de 6 mois dans le cadre du suivi régulier de leur infection chronique par le VIH.
Nous utiliserons un numéro d'inclusion (cf. 10.3) afin d'anonymiser les données extraites du dossier médical informatisé Nadis®.

4 Données recueillies

4.1 Données recueillies concernant l'infection par le chikungunya à la phase aigüe

Pendant la période épidémique pour le chikungunya, les données recueillies lors de chaque visite de suivi pour l'infection chronique par le VIH concerneront l'existence ou non d'un épisode infectieux symptomatique d'allure virale évocateur de chikungunya à la phase aigüe (association de fièvre et arthro/myalgies invalidantes d'apparition brutale).

Dans le cas d'un épisode évocateur de chikungunya, nous limiterons notre recueil à quelques variables descriptives dans le but d'assurer une plus grande homogénéité aux données collectées. En effet, le recueil se fera de façon prospective mais aussi en grande partie de façon rétrospective avec d'importants biais de mémorisation attendus à plusieurs mois de la survenue de l'épisode. Ainsi devant un tableau clinique compatible, le clinicien précisera la date de début des symptômes, le mode de prise en charge (ambulatoire sans recours aux soins, ambulatoire avec consultation du médecin traitant ou maison médicale de garde, hospitalière avec passage aux urgences/filière dédiée, hospitalisation en service de médecine conventionnelle, hospitalisation dans un service de soins intensifs/réanimation) et précisera si nécessaire le caractère atypique de l'épisode dans un texte libre. Une demande de sérologie Chikungunya sera prescrite dans le cadre des soins courants lors du prochain bilan systématique de suivi pour l'infection chronique par le VIH.

Le recueil des données cliniques se fera lors de la consultation médicale sur le dossier patient informatisé Nadis® et sur un cahier d'observation électronique. Les données du dossier Nadis seront extraites et exportées dans le cahier d'observation électronique.
Les résultats sérologiques (pré/post épidémiques) seront également saisis dans le dossier médical informatisé du patient (Nadis®), puis importées dans le cahier d'observation électronique.

4.2 Données recueillies concernant l'infection par le chikungunya à la phase chronique

Pendant les 18 mois suivant l'apparition d'un tableau évocateur d'un épisode aigue d'infection par le chikungunya., les données recueillies lors de chaque visite de suivi pour l'infection chronique par le VIH concerneront l'existence ou non de rhumatismes inflammatoires chroniques évocateurs de chikungunya à la phase chronique selon les quatre profils individualisés dans la littérature (polyarthrite rhumatoïde, rhumatisme psoriasique avec ou sans atteinte axiale, périostites des poignets et des chevilles et douleurs/gonflements articulaires non spécifiques). Devant un tableau évocateur, le patient sera adressé en consultation à un rhumatologue dont l'expertise confirmera ou non si les symptômes sont compatibles avec une infection par le chikungunya à la phase chronique.

5 Critères de jugement

5.1 Critères de jugement principal

Présence ou non d'anticorps d'IgG spécifiques du chikungunya dans le sérum/plasma prélevé à la fin de l'épidémie.

5.2 Critères de jugement secondaire

- Existence d'un épisode évocateur d'infection symptomatique par le chikungunya à la phase aigüe dans l'intervalle d'une séroconversion entre 2 sérums consécutifs testés (mesures de séroprévalence rétrospectives chez les patients séropositifs pour le chikungunya en période post épidémique).
- Existence d'un tableau clinique évocateur d'infection chronique par le chikungunya.

6 Déroulement et organisation de l'enquête

6.1 Calendrier de la recherche

Début théorique de début des inclusions: 01/01/2015
Durée de la période d'inclusion : 8 mois
Fin théorique de la période d'inclusion : 31/08/2015
Date de fin estimée de la recherche : 18 mois suivants le début des symptômes de chikungunya du dernier patient symptomatique inclus.

6.2 Recrutement des centres investigateurs

Les services de Maladies infectieuses et Tropicales du CHU de Martinique (Martinique) et du CHU de Pointe-à-Pitre/Abymes (Guadeloupe), assurent le suivi de la quasi-totalité des patients infectés par le VIH dans leur territoire respectif.
Les accords du chef de service et du directeur de l'établissement hospitalier du CHU Martinique seront préalablement recherchés afin de s'assurer de leur participation à l'étude. Il sera demandé aux investigateurs de chaque centre, la signature d'une charte d'adhésion (accord de participation) à l'étude.

6.3 Modalité d'inclusion et recueil du consentement, du questionnaire et des échantillons biologiques

6.3.1 Tirage au sort

Pour chaque centre participant, nous avons établi une sélection de patients ayant eu un prélèvement biologique avec dosage de la charge virale VIH dans les 6 mois précédent l'épidémie de chikungunya.
Ce listing de patients obtenu a été organisé de façon à ce que les patients présélectionnés soient répartis selon leur classe d'âge (18-34 ans, 35-44 ans, 45-54 ans, 55-64 ans et ≥ 65 ans) et leur sexe.

Dans un second temps, pour chaque centre participant, un tirage au sort a été effectué pour chaque classe d'âge et par sexe. Le nombre de patients tiré au sort est proportionnel aux données du dernier recensement réalisé par l'INSEE en 2011 par territoire, par sexe et classe d'âge multiplié par 3 en prévision des éventuels tubes non exploitables (manquants, quantité insuffisante…) pour la mesure pré épidémique et de refus de participation à l'étude.

6.3.2 Rôle des Médecins investigateurs

6.3.2.1 Information et recueil du consentement éclairé

Pour chaque personne potentiellement éligible, le médecin investigateur présentera le contexte et les objectifs de l'étude a l'aide du document d'information mis à sa disposition, et reverra les critères d'inclusion et de non inclusion . Le médecin investigateur répondra à toutes les questions du patient concernant l'objectif, la nature des contraintes, les risques prévisibles, ses droits ,sa sécurité et les bénéfices attendus de la recherche.
Si la personne accepte de participer à l'enquête, le médecin investigateur recueillera son consentement volontaire, libre et éclairé au prélèvement d'une quantité de 5ml de sang en dehors des soins courants dans les six mois qui suivront la fin de l'épidémie de chikungunya dans leur territoire (date arrêtée par le CIRE Antilles Guyane). Un exemplaire du consentement sera remis au patient participant.
Le médecin investigateur remplira également la fiche confidentielle des patients inclus sur le centre.

Les fiches de suivi des prélèvements et les étiquettes destinées aux tubes de prélèvement seront pré-identifiées avec un numéro d'inclusion unique. Ce numéro d'inclusion comportera une lettre identifiant le centre participant et un numéro d'incrément correspondant à l'ordre d'inclusion des patients dans le centre

6.3.2.2 Saisie des données cliniques

Les données cliniques des patients inclus et suspect d'une infection par le chikungunya, seront saisies par les médecins investigateurs ou les collaborateurs médecins (signature de la délégation de fonction) dans leur dossier médical informatisé Nadis® ainsi que sur l'eCRF de l'étude.

6.3.2.3 Collecte des plasmas/sérums

- **Collecte des plasmas :**

  Plasmas obtenus avant l'épidémie de chikungunya :
  Les plasmas des patients tirés au sort et obtenus à l'occasion d'un prélèvement (charge virale VIH réalisée dans le cadre du suivi biologique régulier) dans les 6 mois précédent l'épidémie de chikungunya seront conservés au laboratoire de virologie de référence. Seuls les plasmas des patients effectivement inclus dans la recherche seront utilisés pour effectuer le test sérologique initial (dosage IgG).

  Plasmas obtenus durant l'épidémie de chikungunya :
  Les plasmas des patients pour lesquels le test sérologique final sera positif seront exploités rétrospectivement à partir de prélèvements effectués depuis l'inclusion des patients jusqu'à la fin de l'épidémie (charge virale VIH réalisée dans le cadre du suivi biologique régulier).

- **Collecte des sérums :**

  Sérums obtenus après l'épidémie de chikungunya :
  Durant les 6 premiers mois suivant la fin officielle de l'épidémie (date arrêtée par le CIRE Antilles Guyane), les sérums de l'ensemble des patients inclus dans l'étude seront collectés à l'occasion soit par un prélèvement lié au suivi de leur infection chronique par le VIH, soit par un prélèvement hors du suivi habituel en convoquant à nouveau les patients pour un prélèvement dédié. Il sera utilisé pour effectuer le test sérologique final de l'étude (dosage IgG).

6.4 Sérologies

6.4.1 Organisation des analyses

Le laboratoire de virologie du CHU de Martinique réalisera les tests sérologiques (IgG) des patients inclus au CHU de Martinique.
Le laboratoire de virologie CERBA réalisera les tests sérologiques (IgG) des patients inclus au CHU de Pointe à Pitre, pour les mesures pré épidémiques.
Le laboratoire de virologie du CHU de Pointe à Pitre réalisera les tests sérologiques (IgG) des patients inclus au CHU de Pointe à Pitre, pour la mesure post épidémique.

6.4.2 Réalisation des analyses sérologiques

Les tests sérologiques de détection des anticorps spécifiques (IgG) du chikungunya seront réalisés selon le protocole développé par le CNR arbovirus.

Analyse réalisée en aveugle vis à vis de l'âge et du sexe de la personne chez qui le prélèvement aura été réalisé.

6.4.3 Résultats des analyses sérologiques

Les résultats des analyses sérologiques identifiés par le numéro anonyme (numéro d'inclusion), seront saisis par les médecins investigateurs, les collaborateurs des médecins ou une personne sous la responsabilité et la validation de l'investigateur (signature de la délégation de fonction).

6.5 Rendu des résultats individuels

Les résultats des sérologies vis à vis des anticorps spécifiques du chikungunya seront ajoutés au dossier

source des participants à la recherche. Les personnes incluses dans l'étude auront été informées de la possibilité d'obtenir les résultats des analyses sérologique pour le chikungunya après la fin de l'étude. Les participants qui manifesteront le désir de connaitre leurs résultats d'analyses, recevront l'information par courrier ou directement du médecin en charge de leur suivi médical.

7 Gestion des évènements indésirables

7.1 Définitions

- **Evénement indésirable (EI)** : toute manifestation nocive survenant chez une personne qui se prête à une recherche biomédicale que cette manifestation soit liée ou non à la recherche.
- **Evénement indésirable grave (EIG)** : la gravité est définie par l'une des constatations suivantes :
  - Décès
  - Mise en jeu du pronostic vital (menace vitale immédiate, au moment de l'évènement, et ce, indépendamment des conséquences qu'aurait une thérapeutique correctrice ou palliative)
  - Incapacité ou handicap important ou durable
  - Hospitalisation
  - Prolongation d'hospitalisation
  - Malformation/anomalie congénitale
  - Evènement potentiellement grave (évènement clinique indésirable ou résultat de laboratoire à caractère grave ou considéré comme tel par l'investigateur)
- **Effet indésirable :** Tout événement indésirable imputable aux procédures du protocole devient un effet indésirable.
- **Effet indésirable inattendu :** Tout effet indésirable dont la nature, la sévérité ou l'évolution ne concorde pas avec les informations relatives aux produits, actes pratiqués, et méthodes utilisées au cours de la recherche. L'évaluation du caractère inattendu d'un effet indésirable se fait sur la base des informations décrites dans le protocole ou la brochure pour l'investigateur, relatives notamment, le cas échéant, aux actes et méthodes pratiqués au cours de la recherche ou aux produits faisant l'objet de la recherche ou utilisés pour les besoins de la recherche.
- **Fait nouveau :** Nouvelle donnée de sécurité, pouvant conduire à une réévaluation du rapport des bénéfices et des risques de la recherche, ou qui pourrait être suffisant pour envisager des modifications des documents relatifs à la recherche, de la conduite de la recherche ainsi que, le cas échéant, dans l'utilisation du produit. (Arrêté du 24 mai 2006**)**

7.2 Rôle de l'investigateur et notification des évènements indésirables, grossesse, fait nouveau de sécurité

L'investigateur évalue chaque événement indésirable au regard de sa gravité. Il doit notifier à l'unité de vigilance, sans délai à partir du jour où il en a connaissance, les événements indésirables graves identifiés comme requérant une notification immédiate (voir paragraphe 7.3) ou tout fait nouveau, s'il survient :

- à partir de la date de signature du consentement,
- pendant toute la durée de suivi du participant prévue par la recherche,
- après la fin du suivi du participant prévue par la recherche, lorsqu'il est susceptible d'être dû à la recherche.

7.3 Evènement indésirable grave attendus

.
Les événements indésirables graves attendus au cours de cette recherche sont :
- **les effets indésirables graves des traitements pour la prise en charge de l'infection par le VIH et/ou de l'infection par le CHIKUNGUNYA, tels que listés dans les résumés des caractéristiques des produits administrés.**
- **les complications induites par les pathologies à l'étude (HIV, CHIKUNGUNYA).**

Ces événements indésirables graves ne doivent pas être rapportés immédiatement à l'USV du promoteur, mais doivent être rapportés dans le cahier d'observation des patients.

Tableau de modalité de notification :

Type d'événement	Modalités de notification	**Délai de notification à l'USV**
**EI + EIG ne nécessitant pas une notification immédiate (paragraphe 7.3)**	Dans le dossier médical informatisé Nadis®	**Pas de notification**
**EIG**<br>A l'exception de ceux listés comme ne nécessitant pas une notification immédiate **(paragraphe 1.3)**	Dans le dossier médical informatisé Nadis®<br>+ Formulaire de déclaration d'EIG<br>Rapport écrit si nécessaire	**Notification immédiate**
**Grossesse**	Formulaire de notification d'un cas de grossesse	**Notification immédiate**
**Fait nouveau**	Formulaire de notification d'un fait nouveu	**Notification immédiate**

Tous ces événements devront être suivis jusqu'à la **complète résolution ou consolidation**.

Pour tout rapport transmis à l'USV, un complément d'information (fiche de notification complémentaire) concernant l'évolution de l'événement, si elle n'est pas mentionnée dans le premier rapport, sera envoyée à l'unité par l'investigateur.

Modalités de notification
Les EIG, doivent être déclarés immédiatement (dans les **24 heures** de sa survenue ou dès la prise de connaissance par l'investigateur, par fax à l'unité de sécurité et de vigilance de CHU de Bordeaux (au **05.57.82.08.34**) ou par mail à vigilance.essais-cliniques@chu-bordeaux.fr.

7.3.1 Période de notification

L'investigateur a la responsabilité de notifier et de rapporter tous les EIG survenant pendant toute la période de l'étude :
- ✓ à partir de la date de signature du consentement,
- ✓ pendant toute la durée de suivi du participant prévue par l'étude

7.3.2 Rôle de l'unité de sécurité et de vigilance du CHU de Bordeaux

L'Unité de sécurité et de vigilance du CHU de Bordeaux se chargera de suivre et de centraliser les notifications immédiates d'évènements indésirables graves. Une copie des formulaires de déclaration initiale et de suivi d'EIG ou de grossesse sera envoyée pour information par l'unité de sécurité et de vigilance au responsable de la responsable d'étude clinique en charge de la recherche.

Après évaluation de la gravité et du lien de causalité de ces évènements, l'Unité de sécurité et de vigilance déclarera les EIG liés aux procédures de l'étude et inattendu et les données importantes de sécurités survenues au cours de la recherche, dans les délais réglementaires

- à l'ANSM (articles L. 1123-10 du code de santé publique),
- au Comité de Protection des Personnes concerné (article R. 1123-53 du CSP),

7.3.3 Rapport annuel de sécurité

A la date d'anniversaire de la première inclusion, l'unité de vigilance rédige un rapport de sécurité comprenant :

- la liste des effets indésirables graves susceptibles d'être liés à la recherche incluant les effets graves attendus et inattendus.
- une analyse concise et critique de la sécurité des participants se prêtant à la recherche.

A la date d'anniversaire de la première inclusion, la cellule promotion de la DRCI du CHU de Martinique contacte par mail l'unité de vigilance afin de lui signifier la nécessité de rédiger le rapport annuel de sécurité de l'essai. L'unité de vigilance réalise ce rapport dans le respect des exigences réglementaires en vigueur et transmet le rapport finalisé à l'ANSM et au CPP dans les 60 jours suivant la date anniversaire de la première inclusion fournie par le promoteur (DRCI du CHU de Martinique).

8 Aspects statistiques

8.1 Constitution de l'échantillon à étudier

8.1.1 Calcul du nombre de sujets nécessaires

Le calcul du nombre de sujets nécessaires est basé sur le taux d'attaque estimé de l'épidémie de chikungunya qui a sévi à la Réunion entre 2005 et 2006 soit 38,2%(11).
Le nombre de sujets nécessaire pour l'estimation d'une séroprévalence post épidémique à 38% pour un intervalle à 95% est de 362.

8.1.2 Répartition du nombre de sujets nécessaires

Les 362 patients nécessaires ont été proportionnellement répartis par territoire, par sexe et classe d'âge selon les données du dernier recensement réalisé par l'INSEE en 2011 (17.3 Annexe 3).

8.1.3 Répartition géographique des centres recruteurs

Les services de Maladies infectieuses et Tropicales du CHU de Martinique (Martinique), du CHU de Pointe-à-Pitre/Abymes (Guadeloupe), assurent le suivi de la quasi-totalité des patients infectés par le VIH aux Antilles françaises.
Cette exhaustivité assure une représentativité de la population antillaise en termes démographiques et géographiques au sein de notre échantillon de patients. La participation de l'ensemble de ces centres est donc indispensable à cette étude de séroprévalence basée sur une cohorte de patient atteint par le VIH.

8.2 Méthodes statistiques employées

L'estimation du taux de séroconversion de l'épidémie de chikungunya se fera par la mesure de la prévalence des IgG positives pour le chikungunya en période post épidémique. Cette prévalence sera estimée après validation et le gel de base final des données avec un intervalle à 95% de façon globale (ensemble de l'échantillon), par classe d'âge et par territoire (Martinique et Guadeloupe).

La prévalence des formes asymptomatiques et des formes chroniques sera estimée avec un intervalle de confiance à 95% sur l'ensemble de la population étudiée et par classe d'âge.

Les données seront analysées sur le logiciel Stata® 13 par le Pr André Cabié (pôle Martinique), médecin investigateur principal.

9 Surveillance de la recherche

Le comité scientifique sera constitué par l'ensemble des investigateurs principaux de chaque centre et de personnalités qualifiées pour leur expertise sur le sujet de l'enquête. Il sera présidé par le coordinateur de l'enquête. La composition du comité scientifique est indiquée en annexe (17.2 Annexe 2).
Ce comité aura pour mission de valider le protocole de l'enquête et d'en vérifier l'application. Notamment il s'assurera que les investigateurs proposent bien l'enquête à l'ensemble des patients éligibles, que le sujet éligible n'est inclus que s'il a signé au préalable le formulaire de consentement éclairé et volontaire. Il y étudiera éventuellement les modifications à y apporter en cours d'enquête. Il centralisera les résultats en fin d'enquête, les analysera et prendra en charge leur publication dans une revue scientifique après les avoir soumis à l'ensemble des investigateurs.

L'attaché de recherche clinique conformément aux bonnes pratiques cliniques et épidémiologiques, aidera l'investigateur à la conduite de l'enquête. Il lui rendra visite avant, pendant et après l'enquête et devra s'acquitter des procédures suivantes :

- S'assurer que l'investigateur et le personnel ont été correctement informés et formés sur le protocole de recherche et ses modalités pratiques,
- Contrôler l'adhésion et le respect du protocole,
- S'assurer du recueil des consentements signés pour tous les patients inclus dans le protocole,

La recherche prévoit comme procédures supplémentaires un prélèvement sanguin. Il n'y a pas de produit à l'étude. Le seul risque a priori entraîné par l'étude pour le patient est celui lié à la réalisation d'un prélèvement sanguin pour le test sérologique (IgG) final. Il est donc considéré comme minime et ne rend pas la constitution d'un comité indépendant de surveillance nécessaire.

10 Droits d'accès aux données et documents sources

10.1 ACCES AUX DONNEES

Le promoteur est chargé d'obtenir l'accord de l'ensemble des parties impliquées dans la recherche afin de garantir l'accès direct à tous les lieux de déroulement de la recherche, aux données sources, aux documents sources et aux rapports dans un but de contrôle de qualité et d'audit par le promoteur.
Les investigateurs mettront à disposition les documents et données individuelles strictement nécessaires au suivi, au contrôle de qualité et à l'audit de la recherche, à la disposition des personnes ayant un accès à ces documents conformément aux dispositions législatives et réglementaires en vigueur (articles L.1121-3 et R.5121-13 du code de la santé publique).

10.2 DONNEES SOURCES

Tout document ou objet original permettant de prouver l'existence ou l'exactitude, d'une donnée ou d'un fait enregistré au cours de la recherche est défini comme document source. Le document source principal est le dossier médical informatisé (Nadis®) de chaque patient participant.

10.3 CONFIDENTIALITE DES DONNEES

Conformément aux dispositions législatives en vigueur (articles L.1121-3 et R.5121-13 du code de la santé publique), les personnes ayant un accès direct aux données sources prendront toutes les précautions nécessaires en vue d'assurer la confidentialité des informations relatives aux médicaments expérimentaux, aux recherches, aux personnes qui s'y prêtent et notamment en ce qui concerne leur

identité ainsi qu'aux résultats obtenus. Ces personnes, au même titre que les investigateurs eux-mêmes, sont soumises au secret professionnel.
Pendant la recherche ou à son issue, les données recueillies sur les personnes qui s'y prêtent et transmises au promoteur par les investigateurs (ou tout autre intervenant spécialisé) seront rendues anonymes. Le code d'anonymisation du patient dans l'enquête comportera :

- un code à 1 lettre : F pour Fort-de-France et P pour Pointe-à-Pitre/Abymes
- le numéro d'inclusion dans le centre (numéro à 3 chiffres) par ordre d'inclusion dans les centres
- un code patient de 2 lettres : première lettre du nom, première lettre du prénom

Exemple :

F-001 IC (pour le 1er patient inclus dans le centre F)
P-003 PM (pour le 3ème patient inclus dans le centre P)

Le promoteur s'assurera que chaque personne qui se prête à la recherche a donné :
- son accord par écrit (consentement Nadis®) pour l'accès aux données individuelles le concernant.
- son accord écrit de participation volontaire à l'étude.

11 Contrôle et assurance qualité

11.1 Consignes pour le recueil des données

Toutes les informations requises par le protocole doivent être consignées sur les cahiers d'observation et une explication doit être apportée pour chaque donnée manquante. Les données devront être recueillies au fur et à mesure qu'elles sont obtenues, et transcrites dans ces cahiers électroniques (eCRF)..
Les données erronées relevées sur les cahiers d'observation seront clairement identifiés à l'aide du système de traçabilité de corrections des données présent dans l'eCRF (la donnée modifiée, la date, la justification de la modification par l'investigateur ou la personne autorisée seront ainsi tracés).
Le recueil des données sera effectué à l'aide de cahiers d'observation standardisés électroniques accessibles à tous les centres par l'intermédiaire de la plate-forme Clinsight® du CIC Antilles-Guyane. Les données anonymisées seront saisies par les ARC (attachés de recherche clinique) et TEC (technicien de recherche clinique) à partir d'un cahier d'observation papier ou d'un dossier médical informatisé sous la responsabilité de l'investigateur.

11.2 Suivi de la recherche

Le suivi de la recherche sera assuré par un technicien de recherche clinique. Il sera chargé, auprès de l'investigateur coordonnateur, de :

- la logistique et la surveillance de la recherche,
- l'établissement des rapports concernant son état d'avancement,
- la vérification de la mise à jour des données dans Nadis® (demande d'informations complémentaires, corrections…)

Il travaillera conformément aux procédures opératoires standardisées du CIC Antilles - Guyane, en collaboration avec l'attaché de recherche clinique délégué par le CIC Antilles - Guyane.

11.3 Contrôle de Qualité

Un Attaché de Recherche Clinique (ARC) du CIC Antilles Guyane s'assurera de la bonne réalisation de l'enquête, du recueil des données, de leur documentation, enregistrement et rapport, en accord avec les procédures opératoires standards mises en application au sein du CIC Antilles Guyane et conformément aux dispositions législatives et réglementaires en vigueur.
La nature et la fréquence du monitoring seront établies selon la grille monitoring/risque definie selon les procédures opératoires standardisées du CIC Antilles Guyane. Tout au long de l'enquête, ce plan sera adapté en fonction des besoins des centres investigateurs.

L'ARC contrôlera, tout au long de l'enquête :
- Les données recueillies
- Les consentements signés de tous les patients inclus

A ce titre, l'investigateur s'engage à mettre à la disposition de l'ARC lors de ses visites de monitoring :
- Les dossiers sources des patients
- Les formulaires de consentement des patients inclus

Les visites effectuées par les ARC et selon les procédures opératoires standardisées du CIC Antilles Guyane permettront d'évaluer :
- La protection des personnes
- La fiabilité des données par rapport aux documents sources
- La conformité de l'enquête par rapport au protocole, aux Bonnes Pratiques Cliniques, à la déontologie et bonnes pratiques en Epidémiologie, et à la législation en vigueur en matière de recherche clinique.

Au terme de ce contrôle qualité, un rapport de monitoring sera rédigé par l'ARC et remis aux médecins délégués du CIC qui prendront des directives en fonctions des conclusions de ce rapport.

12 Considérations éthiques et réglementaires

12.1 Cadre réglementaire de l'enquête

L'enquête «ChikVIH» est une recherche biomédicale ne portant pas sur des produits de santé mentionnés à l'article L 5311-1 du code de la santé publique. Cette recherche entre donc dans le cadre législatif de la recherche biomédicale. Elle comporte une estimation du taux de séroconversion de la première épidémie de chikungunya aux Antilles Françaises au sein d'un échantillon représentatif de la population générale antillaise française répartie par territoire (Martinique et Guadeloupe). La population source utilisée lors du tirage au sort est une cohorte de patients suivis pour une infection chronique par le VIH dans un des centres participants. Cette estimation est réalisée à partir de prélèvements sanguins (article L1221-8-1 du code de la santé publique).

12.2 Engagement du promoteur et des investigateurs

Le promoteur et les investigateurs s'engagent à ce que cette recherche soit réalisée en conformité avec la loi n°2004-806 du 9 août 2004, ainsi qu'en accord avec les Bonnes Pratiques Cliniques (I.C.H. version 4 du 1er mai 1996 et décision du 24 novembre 2006), et la déclaration d'Helsinki (Principes éthiques applicables aux recherches médicales sur des sujets humains, Fortaleza 2013).

La recherche sera conduite conformément au présent protocole. Hormis dans les situations d'urgence nécessitant la mise en place d'actes thérapeutiques précis, les investigateurs s'engagent à respecter le protocole en tous points en particulier en ce qui concerne le recueil du consentement. Pour ce faire, lors de la mise en place, un exemplaire de l'engagement scientifique daté et signé, par chaque investigateur de chaque centre participant sera remis au représentant du promoteur.

12.3 Comité de Protection des Personnes et ANSM

Le protocole, le formulaire d'information et l'attestation de consentement de l'étude ont été soumis pour avis au Comité de Protection des Personnes Sud-Ouest Outre-Mer III, le 29/11/2014.

La notification de l'avis favorable du CPP a été transmise au promoteur de l'étude et à l'Autorité compétente. Une demande d'autorisation a été adressée par le Promoteur à l'ANSM et a reçu un avis favorable pour débuter l'étude le 05/11/2014.

La version (2.0) du protocole a reçu l'avis favorable CPP SOOM III le 25/03/2015 (MS 1) et une autorisation de l'ANSM le 12/03/2015 (MSA 1).
La présente version (3.0) du protocole a reçu l'avis favorable CPP SOOM III le JJ/MM/AAAA (MS 2) et une autorisation de l'ANSM le JJ/MM/AAAA (MSA 2).

12.4 Amendement au protocole

En cas de modification substantielle apportée au protocole par l'investigateur coordonnateur, elle sera approuvée par le promoteur. Ce dernier devra obtenir préalablement à sa mise en œuvre un avis favorable du CPP et une autorisation de l'ANSM dans le cadre de leurs compétences respectives.

Les modifications non substantielles, c'est-à-dire celles n'ayant pas d'impact significatif sur quelque aspect de la recherche que ce soit, sont communiquées au CPP à titre informatif.

Tous les amendements au protocole devront être portés à la connaissance de tous les investigateurs qui participent à la recherche. Comme prévu dans l'engagement scientifique au préalablement signé, les investigateurs s'engagent à en respecter le contenu.

Tout amendement qui modifie la prise en charge des patients ou des bénéfices, risques et contraintes de la recherche fera l'objet d'une nouvelle note d'information et d'un nouveau formulaire de recueil du consentement dont le recueil se fera selon la procédure cité ci-dessous.

12.5 Information du patient et formulaire de consentement éclairé écrit

Les patients seront informés de façon complète et loyale, en des termes compréhensibles, clairs sur les objectifs et les-contraintes de l'étude, des risques éventuels encourus, des mesures de surveillance et de sécurité nécessaires, de la possibilité d'obtenir les résultats des tests biologiques (IgG) spécifiques du chikungunya et de leurs droits de refuser de participer à l'étude ou de la possibilité de se rétracter à tout moment.

Toutes ces informations figurent sur un formulaire d'information et de consentement remis au patient. Le consentement libre, éclairé et écrit du patient sera recueilli par l'investigateur, ou un médecin qui le représente avant l'inclusion définitive dans l'étude (cf. 6.3.2). Une copie du formulaire d'information et de consentement signé par les deux parties sera remise au patient, l'investigateur en conservera l'original. Une copie sera placée en fin d'étude dans une enveloppe inviolable scellée regroupant l'ensemble des formulaires de consentement, celle-ci sera archivée par le promoteur.

12.6 Assurances

Le CHU de Martinique promoteur de cette recherche, a souscrit un contrat d'assurance en responsabilité civile auprès de la société SHAM, conformément aux dispositions des articles L1221-8-1 et L1121-10 du code de la santé publique.

12.7 Traitement informatique des données

Les données enregistrées à l'occasion de cette recherche font l'objet d'un traitement informatisé par le CIC Antilles-Guyane dans le respect de la loi n°78-17 du 6 janvier 1978 relative à l'informatique, aux fichiers et aux libertés, modifiée par la loi 2004-801 du 6 août 2004.

Cette recherche entre dans le cadre de la « Méthodologie de référence » (MR-001) en application des dispositions de l'article 54 alinéas 5 de la loi du 6 janvier 1978 modifiée relative à l'information, aux fichiers et aux libertés. Ce changement a été homologué par décision du 5 janvier 2006. Le CHU de Martinique promoteur de la recherche, a signé un engagement de conformité à cette « Méthodologie de référence » en date du 19 Décembre 2008, sous le N° de déclaration: **1665694**

13 Financement de la recherche

Cette recherche sera financée par le CHU de Martinique dans le cadre du CONTRAT DE PROJETS ETAT – REGION - DEPARTEMENT MARTINIQUE: Dengue et Viroses tropicales
Les dépenses se limiteront à la réalisation des tests sérologiques prévus par la recherche.

14 Traitement des données et conservation des documents et des données relatives à la recherche

14.1 Traitement des données

Pour chaque participant à l'étude «ChikVIH» est tenu un cahier d'observation papier et/ou un cahier d'observation électronique selon la visite et les conditions pratiques de réalisation de la consultation. Il sera rempli par l'investigateur du centre ou par un TEC sous la responsabilité et la validation de l'investigateur.
Le cahier d'observation sera ensuite saisi par voie électronique sur un portail Internet sécurisé par l'intermédiaire du logiciel Capture System®.
Capture System® est un logiciel de gestion de bases de données cliniques, utilisant la base de données Oracle®. Le design et l'apparence de la base seront inchangés par rapport au CRF, les fenêtres de saisie étant des images des pages du CRF. Les serveurs hébergeant les bases de données sont protégés par un pare-feu, les ports de communication avec les serveurs de pages sont filtrés et la connexion est cryptée.
Cs-Online®, module de saisie en ligne des données est intégré à Capture System®. La saisie sera simple et les données transmises sont cryptées en mode SSL jusqu'à 128 bits, directement par un navigateur. Les données sont saisies après s'être connecté sur la base avec un nom d'utilisateur et un mot de passe unique pour chaque utilisateur, et lui donnant certains droits de visualisation et de modification suivant son profil.
L'historique de chaque donnée (avec l'ensemble des modifications, le nom de l'utilisateur et la date de modification) peut être visualisé. Le logiciel Capture System respecte la norme 21 CFR Part 11, ainsi que la norme concernant la sécurité des systèmes informatisés.
Une fois saisies, les données peuvent être exportées au format SAS®, SPSS®, STATVIEW®, EXCEL®, TXT.
Les tests de cohérence seront exécutés directement sous Capture System. Ils donneront lieu à émission de demandes de corrections (queries/requêtes).
Les données sont validées conformément au plan de data management défini conjointement entre l'investigateur coordinateur et le CIC (méthodologiste, data manager et statisticien).

Les données sont validées conformément au plan de data management défini conjointement entre l'investigateur coordinateur et le CIC (méthodologiste, data manager et statisticien).

Le processus de gel/dégel des données est réalisé conformément à la procédure mise en place au CIC Antilles Guyane

14.1 Conservation des documents relatifs à la recherche

Les documents suivants relatifs à cette recherche sont archivés conformément aux Bonnes Pratiques Cliniques :

Par les médecins investigateurs :

- pour une durée de 15 ans suivant la fin de la recherche

- Le protocole et les amendements éventuels au protocole
- Données anonymes extraites du dossier médical informatisé Nadis® et cahiers d'observation
- Les dossiers sources des participants ayant signé un consentement Nadis® et un consentement pour la participation à la recherche.
- Tous les autres documents et courriers relatifs à la recherche

- pour une durée de 30 ans suivant la fin de la recherche

- L'exemplaire original des consentements éclairés (Nadis® et pour la participation à la recherche) signés des participants

Tous ces documents sont sous la responsabilité de l'investigateur pendant la durée réglementaire d'archivage.

Par le promoteur :

- pour une durée de 15 ans suivant la fin de la recherche

- Le protocole et les amendements éventuels au protocole
- Données anonymes extraites du dossier médical informatisé Nadis® et les originaux des cahiers d'observation
- Tous les autres documents et courriers relatifs à la recherche

- pour une durée de 30 ans suivant la fin de la recherche

- Un exemplaire des consentements éclairés signés des participants

Tous ces documents sont sous la responsabilité du promoteur pendant la durée réglementaire d'archivage.

Aucun déplacement ou destruction ne pourra être effectué sans l'accord du promoteur. Au terme de la durée réglementaire d'archivage, le promoteur sera consulté pour destruction. Toutes les données, tous les documents et rapports pourront faire l'objet d'audit ou d'inspection.

15 Règles relatives à la publication

15.1 Communications scientifiques

L'analyse des données fournies par les centres investigateurs est réalisée par le CIC Antilles – Guyane. Toute communication écrite ou orale des résultats de la recherche doit recevoir l'accord préalable de l'investigateur coordonnateur et du comité scientifique de l'enquête.
La publication des résultats principaux mentionne le nom du promoteur, de tous les investigateurs ayant inclus ou suivi des patients dans la recherche, des membres du comité scientifique et de la source de financement. Il sera tenu compte des règles internationales d'écriture et de publication (Convention de Vancouver, février 2006).

15.2 Communication des résultats aux patients

Conformément à la loi n°2002-303 du 4 mars 2002, les patients sont informés, à leur demande, des résultats globaux de la recherche.

15.3 Cession des données

Le recueil et la gestion des données sont assurés par le CIC Antilles – Guyane. Les conditions de cession de tout ou partie de la base de données de la recherche sont décidées par le promoteur de la recherche et font l'objet d'un contrat écrit.

16 Références Bibliographiques

1. Pialoux G, Gaüzère B-A, Jauréguiberry S, Strobel M. Chikungunya, an epidemic arbovirosis. Lancet Infect Dis. 2007 May;7(5):319–27.

2. Farnon EC, Sejvar JJ, Staples JE. Severe disease manifestations associated with acute chikungunya virus infection*: Crit Care Med. 2008 Sep;36(9):2682–3.

3. Brouard C, Bernillon P, Quatresous I, Pillonel J, Assal A, De Valk H, et al. Estimated risk of Chikungunya viremic blood donation during an epidemic on Reunion Island in the Indian Ocean, 2005 to 2007. Transfusion (Paris). 2008 Jul;48(7):1333–41.

4. Liumbruno GM, Calteri D, Petropulacos K, Mattivi A, Po C, Macini P, et al. The Chikungunya epidemic in Italy and its repercussion on the blood system. Blood Transfus Trasfus Sangue. 2008 Oct;6(4):199–210.

5. Sissoko D, Moendandze A, Malvy D, Giry C, Ezzedine K, Solet JL, et al. Seroprevalence and risk factors of chikungunya virus infection in Mayotte, Indian Ocean, 2005-2006: a population-based survey. PloS One. 2008;3(8):e3066.

6. Appassakij H, Khuntikij P, Kemapunmanus M, Wutthanarungsan R, Silpapojakul K. Viremic profiles in asymptomatic and symptomatic chikungunya fever: a blood transfusion threat? Transfusion (Paris). 2013 Oct;53(10 Pt 2):2567–74.

7. Bianco C. Dengue and Chikungunya viruses in blood donations: risks to the blood supply? Transfusion (Paris). 2008 Jul;48(7):1279–81.

8. Ramful D, Carbonnier M, Pasquet M, Bouhmani B, Ghazouani J, Noormahomed T, et al. Mother-to-child transmission of Chikungunya virus infection. Pediatr Infect Dis J. 2007 Sep;26(9):811–5.

9. Gérardin P, Barau G, Michault A, Bintner M, Randrianaivo H, Choker G, et al. Multidisciplinary prospective study of mother-to-child chikungunya virus infections on the island of La Réunion. PLoS Med. 2008 Mar 18;5(3):e60.

10. CIRE Antilles Guyane. Alerte “chikungunya” dans les Antilles. Point Épidémio. 2013 Dec 19;(2).

11. Gérardin P, Guernier V, Perrau J, Fianu A, Le Roux K, Grivard P, et al. Estimating Chikungunya prevalence in La Réunion Island outbreak by serosurveys: two methods for two critical times of the epidemic. BMC Infect Dis. 2008;8:99.

12. Van Bortel W, Dorleans F, Rosine J, Blateau A, Rousset D, Matheus S, et al. Chikungunya outbreak in the Caribbean region, December 2013 to March 2014, and the significance for Europe. Euro Surveill Bull Eur Sur Mal Transm Eur Commun Dis Bull. 2014;19(13).

17 Annexes

17.1 Annexe 1 : liste des investigateurs

Code postal*	Investigateur	Adresse	Téléphone, fax, mail
972	Pr. André Cabié	Service de Maladies infectieuses et Tropicales<br>CHU de Martinique,<br>Hôpital Pierre Zobda Quitman<br>BP 632, 97261 Fort-de-France cedex	Tel : 0596 55 23 01<br>Courriel: andre.cabie@chu- fortdefrance.fr
971	Dr. Elodie Curlier	Service de Maladies infectieuses et Tropicales<br>CHU de Pointe-à-Pitre/Abymes,<br>97159 Pointe-à-Pitre, Guadeloupe	Tel: 0590 89 15 45<br>Courriel: elodie.curlier@chu-guadeloupe.fr

(*) 971 : Guadeloupe, 972 : Martinique

17.2 Annexe 2 : composition du comité scientifique de l'étude «ChikVIH»

Nom, Prénom	Spécialité	Lieu d'activité
Pr. André Cabié	Service de maladies infectieuses et tropicales	CHU de Martinique
Pr. Raymond Césaire	Service de Virologie	CHU de Martinique
Pr. Brunon Hoën	Service de maladies infectieuses et tropicales	CHU de Pointe à Pitre
Dr. Elodie Curlier	Service de maladies infectieuses et tropicales	CHU de Pointe à Pitre
Janick Jean-Marie	CIC-EC Antilles-Guyane	CHU de Martinique

17.3 Annexe 3 : Données démographiques (recensement INSEE 2011) et échantillonnage

Recensement INSEE 2011

Source : Insee RP 2011 exploitation principale

Martinique

	Hommes	Femmes	Total
18 à 34 ans	32 833	38 392	71 225
35 à 44 ans	24 378	32 036	56 414
45 à 54 ans	28 734	34 312	63 046
55 à 65 ans	21 811	25 788	47 599
65 ans ou plus	26 596	35 763	62 359
Total	134 352	166 291	300 643

Guadeloupe

	Hommes	Femmes	Total
18 à 34 ans	33 589	38 944	72 533
35 à 44 ans	25 932	34 366	60 298
45 à 54 ans	28 559	33 124	61 683
55 à 65 ans	21 660	25 528	47 188
65 ans ou plus	24 463	32 967	57 430
Total	134 203	164 929	299 132

Répartition des effectifs par centre pour une prévalence à 38% (N=362)

Martinique

	Hommes	Femmes	Total
18 à 34 ans	20	23	43
35 à 44 ans	15	19	34
45 à 54 ans	17	21	38
55 à 65 ans	13	16	29
65 ans ou plus	16	21	37
Total	81	100	181

Guadeloupe

	Hommes	Femmes	Total
18 à 34 ans	20	24	44
35 à 44 ans	16	21	37
45 à 54 ans	17	20	37
55 à 65 ans	13	15	28
65 ans ou plus	15	20	35
Total	81	100	181

17.4 Annexe 4: Avis du CPP Sud Ouest et Outre Mer III & ANSM

COMITÉ DE PROTECTION DES PERSONNES
SUD-OUEST ET OUTRE MER III
Président : Professeur Emmanuel CUNY

DOSSIER ENREGISTRÉ CPP N° : 2014/82
Numéro d'enregistrement : 2014-A01504-43

CENTRE HOSPITALIER UNIVERSITAIRE DE FORT DE FRANCE
13 NOV. 2014
Délégation à la Recherche Clinique et à l'Innovation
COURRIER ARRIVÉ

DESTINATAIRE : CHU de la Martinique
Janick JEAN-MARIE
CIC Antilles-Guyane
Hôpital Pierre Zobda Quitman
CS 90632
97261 Fort-de-France cedex

Vos réf. : étude ChikVIH

Bordeaux, le 5 novembre 2014.

PROMOTEUR : CHU de la Martinique
Hôpital Pierre-Zobda Quitman
CS 90632
97261 Fort-de-France cedex

COORDONNATEUR : Docteur Vincent RONIN
Service des maladies infectieuses e tropicales
CHU de Martinique
CS 90632
97261 Fort-de-France cedex

En date du **29 OCTOBRE 2014**, conformément aux dispositions du Code de la Santé Publique, le Comité de Protection des Personnes Sud-Ouest et Outre-mer III a examiné le protocole de recherche biomédicale ne portant pas sur un produit mentionné à l'article L. 5311-1 du Code de la santé publique intitulé :

"ESTIMATION DU TAUX D'ATTAQUE DE LA PREMIERE EPIDEMIE DE CHIKUNGUNYA AUX ANTILLES FRANCAISE AU SEIN D'UN ECHANTILLON DE PATIENTS SUIVIS POUR UNE INFECTION PAR LE VIH."

Le comité à l'unanimité des membres votants émet un

AVIS FAVORABLE

Remarques :

1° - Justifier l'absence de lien entre une infection VIH et la séroconversion à Chikungunya ;

2° - Page 20/43 du protocole : en quoi les complications des traitements VIH ou Chikungunya constituent-il des effets indésirables graves attendus de cette étude de séroconversion ? Les seuls effets seraient des problèmes de déplacement et de prise de sang.

Le Président du Comité

Professeur Emmanuel CUNY.

Service de Pharmacologie clinique – Groupe Hospitalier Pellegrin – Bât. 1A
Place Amélie Raba Léon – 33076 BORDEAUX CEDEX
TÉL/FAX : 33-(0)5.57.81.76.07 – E-mail : cpp.soom3@u-bordeaux2.fr
Site Internet : www.cpp-soom3.u-bordeaux2.fr

1/2

COMITÉ DE PROTECTION DES PERSONNES
SUD-OUEST ET OUTRE MER III
Président : Professeur Emmanuel CUNY

DOSSIER ENREGISTRÉ CPP N° : 2014/82
Numéro d'enregistrement : 2014-A01504-43

DESTINATAIRE : CHU de la Martinique
Janick JEAN-MARIE
CIC Antilles-Guyane
Hôpital Pierre Zobda Quitman
CS 90632
97261 Fort-de-France cedex

Vos réf. : étude ChikVIH

Bordeaux, le 30 mars 2015.

Avis favorable avec remarques en date du 29 octobre 2014.

PROMOTEUR : CHU de la Martinique
Hôpital Pierre-Zobda Quitman
CS 90632
97261 Fort-de-France cedex

COORDONNATEUR : Docteur André CABIE
Service des maladies infectieuses e tropicales
CHU de Martinique
CS 90632
97261 Fort-de-France cedex

En date du **25 MARS 2015**, conformément aux dispositions du Code de la Santé Publique, le Comité de Protection des Personnes Sud-Ouest et Outre-mer III a examiné la demande de modification substantielle au protocole de recherche biomédicale ne portant pas sur un produit mentionné à l'article L. 5311-1 du Code de la santé publique intitulé :

"ESTIMATION DU TAUX DE SEROPREVALENCE A LA FIN DE LA PREMIERE EPIDEMIE DE CHIKUNGUNYA AUX ANTILLES FRANCAISE AU SEIN D'UN ECHANTILLON DE PATIENTS SUIVIS POUR UNE INFECTION PAR LE VIH."

L'amendement concerne :

- le changement du titre de la recherche ;
- le changement du coordonnateur de l'étude ;
- le changement d'investigateur principal du centre de Guadeloupe ;
- La modification des ctirèes de non inclusion ;
- L'augmentation de la durée d'inclusion ;
- La modification de l'objectif principal ;
- Une modification concernant le déroulement de la recherche.

Le comité à l'unanimité des membres votants émet un

AVIS FAVORABLE A LA MODIFICATION SUBSTANTIELLE N° 1.

Le Président du Comité

Professeur Emmanuel CUNY.

Service de Pharmacologie clinique – Groupe Hospitalier Pellegrin – Bât. 1A
Place Amélie Raba Léon – 33076 BORDEAUX CEDEX
TÉL/FAX : 33-(0)5.57.81.76.07 – E-mail : cpp.soom3@u-bordeaux2.fr
Site Internet : www.cpp-soom3.u-bordeaux2.fr

Fax émis par : +33155873402 ANSM/INFHEP 05-11-14 09:52 Pg: 1/1

ansm
Agence nationale de sécurité du médicament et des produits de santé

CENTRE HOSPITALIER UNIVERSITAIRE DE FORT-DE-FRANCE
12 NOV. 2014
Délégation à la Recherche Clinique et à l'Innovation
COURRIER ARRIVÉ

AUTORISATION D'ESSAI CLINIQUE NE PORTANT PAS SUR UN PRODUIT DE SANTÉ (ESSAI-HPS)

C.H.U. de Martinique
Direction Générale
- 6 NOV. 2014
COURRIER ARRIVÉ

Nombre de pages : 1
(Incluant la page de garde)

Copie Dr GABRIEL + CIC-EC -

Envoi par Télécopie

Date : 05 NOV. 2014

Identifiants de l'essai clinique					
Titre	Estimation du taux d'attaque de la première épidémie de Chikungunya aux Antilles françaises au sein d'un échantillon de patients suivis pour une infection par le VIH				
Promoteur	CHU DE MARTINIQUE			Réf. CPP	Non disponible
Réf. Promoteur	14/B/18	N° ID RCB	2014-A01504-43	Réf. ANSM	141258B-41

Expéditeur	Destinataire (demandeur : nom / société / tél.)
ANSM / Direction Produit INFHEP/ Equipe Equipe maladies infectieuses	Annick CLEMMER<br>CHU DE MARTINIQUE<br>05 96 55 97 75
Dossier suivi par : Joanna Guirao<br>Tél : 33 (0) 1 55 87 38 09 / Fax : 33 (0) 1 55 87 38 09<br>Mel : joanna.guirao@ansm.sante.fr	Fax 05 96 75 50 60

CPP destinataire en copie	Sud-Ouest et Outre Mer III (Bordeaux)	Fax	05.57.81.76.07	Code 10

Vu le code de la santé publique et notamment ses articles L. 1123-8, R. 1123-32 et vu le dossier de demande d'autorisation d'essai clinique adressé à l'Agence nationale de sécurité du médicament et des produits de santé (ANSM) ;

L'autorisation mentionnée à l'article L. 1123-8 du code de la santé publique est accordée pour l'essai clinique cité en objet. Cette autorisation est valable pour toute la durée de l'essai à compter de la date de la présente décision.

Toutefois, conformément à l'article R. 1123-33 du code de la santé publique, la présente autorisation devient caduque si la recherche n'a pas débuté dans un délai d'un an.

Cette autorisation est délivrée, considérant que les examens complémentaires mis en œuvre pour les besoins de la recherche doivent être effectués avec les mêmes mesures de sécurité que celles habituellement recommandées en pratique clinique. Il revient donc aux investigateurs et intervenants de se conformer aux usages en vigueur.

En outre, je vous rappelle notamment que pendant le déroulement de la recherche et pour ce qui concerne l'ANSM :

- toute modification substantielle du dossier initialement soumis doit faire l'objet d'une demande d'autorisation en vertu des articles L. 1123-9 et R. 1123-35 du code de la santé publique ;
- les effets indésirables graves inattendus ainsi que les faits nouveaux susceptibles de porter atteinte à la sécurité des personnes doivent être déclarés en vertu des articles L. 1123-10 et R. 1123-46 du code de la santé publique.

Le chef produits maladies infectieuses
Direction des médicaments anti-infectieux, en hépato-gastro-entérologie, en dermatologie et maladies métaboliques rares

Nathalie MORGENSZTEJN

Je vous demande de transmettre toute demande d'informations complémentaires concernant ce dossier par courriel adressé à la boite : ams-essaiscliniques@ansm.sante.fr. Je vous précise qu'il vous est possible d'utiliser à cet effet le système de messagerie électronique sécurisée Eudralink. Lors de l'envoi de ces dossiers, je vous demande de veiller à reporter dans l'objet du message les mentions suivantes :
- pour les MS transmises à l'Ansm pour information : **MSI/ Réf ANSM du dossier**
- pour les MS soumises pour autorisation ou pour les dossiers mixtes (comportant des modifications soumises pour autorisation et d'autres pour information) : **MSA/ Réf ANSM du dossier**

Si vous ne recevez pas toutes les pages de cette télécopie, veuillez contacter le secrétariat de la Direction Produit INFHEP **/ Equipe** MALINF **au : 33 (0) 1 55 87 34 04.**

Confidentialité
Cette transmission est à l'attention exclusive du(des) destinataires ci-dessus mentionné(s) et peut contenir des informations privilégiées et/ou confidentielles. Si vous n'êtes pas le destinataire voulu ou une personne mandatée pour lui remettre cette transmission, vous avez reçu ce document par erreur et toute utilisation, révélation, copie ou communication de son contenu est interdite. Si vous avez reçu cette transmission par erreur, veuillez nous en informer par téléphone immédiatement et nous retourner le message original par courrier. Merci.

Confidentiality
This transmission is intended to the addressee(s) listed above only and may contain preferential or/and confidential information. If you are not the intended recipient, you are hereby notified that you have received the document by mistake and any use, disclosure, copying or communication of the content of this transmission is prohibited. If you have received this transmission by mistake, please call us immediately and return the original message by mail. Thank you.

code : Q16CDOC004 v01

143/147, bd Anatole France - F-93285 Saint-Denis cedex - tél. +33 (0)1 55 87 30 00 - **www.ansm.sante.fr**

Page 1 sur 1

Fax émis par : +33155873402 ANSM/INFHEP 12-03-15 17:57 Pg: 1/1

ansm

AUTORISATION DE MODIFICATION (S) SUBSTANTIELLE (S) D'ESSAI(S) CLINIQUE(S) NE PORTANT PAS SUR UN PRODUIT DE SANTE (ESSAI(S)-HPS)

Nombre de pages : 1
(Incluant la page de garde)

Envoi par Télécopie **Date :** 12 MARS 2015

**Identifiants de la (des) modification(s) et du (des) essai(s) concerné(s)**			
**Promoteur**	CHU DE MARTINIQUE		
**Réf. Essai(s)**		**Réf. Modification(s)**	
N° ID RCB	Réf. ANSM	Réf. ANSM	Réf. Promoteur (item D.1 du formulaire de demande d'AMS)
2014-A01504-43	141258B-41	141258S-4101	Protocole version 2 du 22/02/2015

**Expéditeur**
ANSM / Direction Produit INFHEP / Equipe maladies infectieuses
Dossier suivi par : Joanna Guirao<br>Tél : 33 (0) 1 55 87 38 09 / Fax : 33 (0) 1 55 87 34 02

**Destinataire** (demandeur : nom / société / tél.)	
Janick JEAN-MARIE<br>CHU de Martinique<br>Tel : 05 96 59 26 97	
Fax	**05 96 75 76 52**

Vu le code de la santé publique et notamment les articles L. 1123-9, R. 1123-37 et vu la ou les autorisations d'essais cliniques délivrées par l'Agence nationale de sécurité du médicament et des produits de santé (ANSM) pour le ou les essais cliniques ci-dessus référencés ;

Vu le dossier de demande d'autorisation de modification(s) substantielle(s) adressé à l'ANSM ;

L'autorisation mentionnée à l'article L. 1123-9 du code de la santé publique est accordée pour la (les) modification(s) substantielle(s) identifiée(s) ci-dessus, pour les aspects relevant de la compétence de l'ANSM.

Le chef produits maladies infectieuses
Direction des médicaments anti-infectieux, en hépato-gastro-entérologie, en dermatologie et maladies métaboliques rares

Nathalie MORGENSZTEJN

Je vous demande de transmettre toute demande d'informations complémentaires concernant ce dossier par courriel adressé à la boite : **hps-essaiscliniques@ansm.sante.fr**. Je vous précise qu'il vous est possible d'utiliser à cet effet le système de messagerie électronique sécurisée Eudralink. Lors de l'envoi de ces dossiers, je vous demande de veiller à reporter dans l'objet du message les mentions suivantes :

- pour les MS transmises à l'ANSM pour information : **MSI / Réf ANSM du dossier** ;
- pour les MS soumises pour autorisation ou pour les dossiers mixtes (comportant des modifications soumises pour autorisation et d'autres pour information) : **MSA / Réf ANSM du dossier**.

Si vous ne recevez pas toutes les pages de cette télécopie, veuillez contacter le secrétariat de la Direction Produit INFHEP / Equipe maladies infectieuses au : 33 (0) 1 55 87 34 04.

Confidentialité
Cette transmission est à l'attention exclusive du(des) destinataires ci-dessus mentionné(s) et peut contenir des informations privilégiées et/ou confidentielles. Si vous n'êtes pas le destinataire voulu ou une personne mandatée pour lui remettre cette transmission, vous avez reçu ce document par erreur et toute utilisation, révélation, copie ou communication de son contenu est interdite. Si vous avez reçu cette transmission par erreur, veuillez nous en informer par téléphone immédiatement et nous retourner le message original par courrier. Merci.

Confidentiality
This transmission is intended to the addressee(s) listed above only and may contain preferential or/and confidential information. If you are not the intended recipient, you are hereby notified that you have received the document by mistake and any use, disclosure, copying or communication of the content of this transmission is prohibited. If you have received this transmission by mistake, please call us immediately and return the original message by mail. Thank you.

143/147, bd Anatole France - F-93285 Saint-Denis cedex - tél. +33 (0)1 55 87 30 00 - **www.ansm.sante.fr** **Page 1 sur 1**

17.6 Annexe 5 : Attestation d'assurance

sham
PARTAGEONS PLUS QUE L'ASSURANCE

CENTRE HOSPITALIER UNIVERSITAIRE
DE FORT DE FRANCE
15 SEP. 2014
Délégation à la Recherche Clinique
et à l'Innovation
COURRIER ARRIVÉ

ATTESTATION D'ASSURANCE

RESPONSABILITE CIVILE
PROMOTEUR DE RECHERCHES BIOMEDICALES

(Loi n° 2004-806 du 9 août 2004 et textes d'application subséquents)

LA SOCIETE HOSPITALIERE D'ASSURANCES MUTUELLES
18, rue Edouard Rochet - 69372 LYON CEDEX 08

atteste que le CENTRE HOSPITALIER UNIVERSITAIRE DE MARTINIQUE
HÔPITAL PIERRE ZOBDA QUITMAN - CS 90632 - **97261 FORT DE FRANCE CEDEX**

a souscrit sous le n° **145494** un contrat d'assurance de la Responsabilité Civile Promoteur de Recherche Biomédicale conforme aux **dispositions** du décret 2006-477 du 26 avril 2006, afin de couvrir les obligations mises à sa charge en application de l'article L.1121-10 du Code de la Santé Publique.

"ChikVIH : Estimation du taux d'attaque de la première épidémie de Chikungunya aux Antilles françaises au sein d'un échantillon de patients suivis pour une infection par le VIH." (DR RONIN)

La garantie prend effet au plus tôt le **8 septembre 2014** et est automatiquement acquise en cas notamment de modifications affectant le nombre de sujets ou la durée de la recherche.

La présente attestation ne constitue toutefois qu'une présomption d'assurance à la charge de la Société avant validation par les autorités compétentes.

Fait à LYON, le **8 septembre 2014**

SOCIETE HOSPITALIERE
D'ASSURANCES MUTUELLES
- 8 SEP. 2014
18, Rue Edouard Rochet - Lyon 8e

Alain JUTTET
Gestionnaire souscription vie des contrats
Direction Etablissements publics de santé

SHAM - Société Hospitalière d'Assurances Mutuelles
18 rue Édouard Rochet - 69372 LYON Cedex 08
Tél : +33 (0)4 72 75 50 25 - Fax : +33 (0)4 72 74 22 32 - www.sham.fr

Société d'Assurance Mutuelle à cotisations fixes.
Entreprise régie par le code des assurances - 779 860 881 RCS Lyon
N° TVA Intracommunautaire FR 79779860881 - RIP 20041 01007 0033305Z038 15

17.7 Annexe 6 : Déclaration d'Helsinki

Principes éthiques applicables à la recherche médicale impliquant des êtres humains Adoptée par la 18e Assemblée générale de l'AMM, Helsinki, Finlande, Juin 1964 et amendée par les 29e Assemblée générale de l'AMM, Tokyo, Octobre 1975 35e Assemblée générale de l'AMM, Venise, Octobre 1983 41e Assemblée générale de l'AMM, Hong Kong, Septembre 1989 48e Assemblée générale de l'AMM, Somerset West (Afrique du Sud), Octobre 1996 52e Assemblée générale de l'AMM, Edimbourg, Ecosse, Octobre 2000 53e Assemblée générale de l'AMM, Washington, Etats Unis, 2002 (ajout d'une note de clarification pour le paragraphe 29) 55e Assemblée générale de l'AMM, Tokyo, Japon 2004 (ajout d'une note de clarification concernant le paragraphe 30) 59e Assemblée générale de l'AMM, Séoul, Corée, Octobre 2008, 64e générale de l'AMM, Fortaleza, Brésil Octobre 2013.

1 Préambule

1. L'Association Médicale Mondiale (AMM) a élaboré la Déclaration d'Helsinki comme un énoncé de principes éthiques applicables à la recherche médicale impliquant des êtres humains, y compris la recherche sur du matériel biologique humain et sur des données identifiables.

La Déclaration est conçue comme un tout indissociable. Chaque paragraphe doit être appliqué en tenant compte de tous les autres paragraphes pertinents.

2. Conformément au mandat de l'AMM, cette Déclaration s'adresse en priorité aux médecins. L'AMM invite cependant les autres personnes engagées dans la recherche médicale impliquant des êtres humains à adopter ces principes.

2 Principes généraux

3. La Déclaration de Genève de l'AMM engage les médecins en ces termes: «La santé de mon patient prévaudra sur toutes les autres considérations » et le Code International d'Ethique Médicale déclare qu'un «médecin doit agir dans le meilleur intérêt du patient lorsqu'il le soigne».
4. Le devoir du médecin est de promouvoir et de sauvegarder la santé, le bien être et les droits des patients, y compris ceux des personnes impliquées dans la recherche médicale. Le médecin consacre son savoir et sa conscience à l'accomplissement de ce devoir.
5. Le progrès médical est basé sur la recherche qui, en fin de compte, doit impliquer des êtres humains.
6. L'objectif premier de la recherche médicale impliquant des êtres humains est de comprendre les causes, le développement et les effets des maladies et d'améliorer les interventions préventives, diagnostiques et thérapeutiques (méthodes, procédures et traitements). Même les meilleures interventions éprouvées doivent être évaluées en permanence par des recherches portant sur leur sécurité, leur efficacité, leur pertinence, leur accessibilité et leur qualité.
7. La recherche médicale est soumise à des normes éthiques qui promeuvent et assurent le respect de tous les êtres humains et qui protègent leur santé et leurs droits.
8. Si l'objectif premier de la recherche médicale est de générer de nouvelles connaissances, cet objectif ne doit jamais prévaloir sur les droits et les intérêts des personnes impliquées dans la recherche.
9. Il est du devoir des médecins engagés dans la recherche médicale de protéger la vie, la santé, la dignité, l'intégrité, le droit à l'auto-détermination, la vie privée et la confidentialité des informations des personnes impliquées dans la recherche. La responsabilité de protéger les personnes impliquées dans la recherche doit toujours incomber à un médecin ou à un autre professionnel de santé et jamais aux personnes impliquées dans la recherche même si celles-ci ont donné leur consentement.
10. Dans la recherche médicale impliquant des êtres humains, les médecins doivent tenir compte des normes et standards éthiques, légaux et réglementaires applicables dans leur propre pays ainsi que des normes et standards internationaux. Les protections garanties par la présente Déclaration aux personnes impliquées dans la recherche ne peuvent être restreintes ou exclues par aucune disposition éthique, légale ou réglementaire, nationale ou internationale.
11. La recherche médicale devrait être conduite de sorte qu'elle réduise au minimum les nuisances éventuelles à l'environnement.
12. La recherche médicale impliquant des êtres humains doit être conduite uniquement par des personnes ayant acquis une éducation, une formation et des qualifications appropriées en éthique et en science. La

recherche impliquant des patients ou des volontaires en bonne santé nécessite la supervision d'un médecin ou d'un autre professionnel de santé qualifié et compétent.
13. Des possibilités appropriées de participer à la recherche médicale devraient être offertes aux groupes qui y sont sous-représentés.
14. Les médecins qui associent la recherche médicale à des soins médicaux devraient impliquer leurs patients dans une recherche uniquement dans la mesure où elle se justifie par sa valeur potentielle en matière de prévention, de diagnostic ou de traitement et si les médecins ont de bonnes raisons de penser que la participation à la recherche ne portera pas atteinte à la santé des patients concernés.
15. Une compensation et un traitement adéquats doivent être garantis pour les personnes qui auraient subi un préjudice en raison de leur participation à une recherche.

3 Risques, contraintes et avantages

16. Dans la pratique médicale et la recherche médicale, la plupart des interventions comprennent des risques et des inconvénients.

Une recherche médicale impliquant des êtres humains ne peut être conduite que si l'importance de l'objectif dépasse les risques et inconvénients pour les personnes impliquées.
17. Toute recherche médicale impliquant des êtres humains doit préalablement faire l'objet d'une évaluation soigneuse des risques et des inconvénients prévisibles pour les personnes et les groupes impliqués, par rapport aux bénéfices prévisibles pour eux et les autres personnes ou groupes affectés par la pathologie étudiée.

Toutes les mesures destinées à réduire les risques doivent être mises en œuvre. Les risques doivent être constamment surveillés, évalués et documentés par le chercheur.
18. Les médecins ne peuvent pas s'engager dans une recherche impliquant des êtres humains sans avoir la certitude que les risques ont été correctement évalués et pourront être gérés de manière satisfaisante.

Lorsque les risques s'avèrent dépasser les bénéfices potentiels ou dès l'instant où des conclusions définitives ont été démontrées, les médecins doivent évaluer s'ils continuent, modifient ou cessent immédiatement une recherche.

4 Populations et personnes vulnérables

19. Certains groupes ou personnes faisant l'objet de recherches sont particulièrement vulnérables et peuvent avoir une plus forte probabilité d'être abusés ou de subir un préjudice additionnel.

Tous les groupes et personnes vulnérables devraient bénéficier d'une protection adaptée.
20. La recherche médicale impliquant un groupe vulnérable se justifie uniquement si elle répond aux besoins ou aux priorités sanitaires de ce groupe et qu'elle ne peut être effectuée sur un groupe non vulnérable. En outre, ce groupe devrait bénéficier des connaissances, des pratiques ou interventions qui en résultent.

5 Exigences scientifiques et protocoles de recherche

21. La recherche médicale impliquant des êtres humains doit se conformer aux principes scientifiques généralement acceptés, se baser sur une connaissance approfondie de la littérature scientifique, sur d'autres sources pertinentes d'informations et sur des expériences appropriées en laboratoire et, le cas échéant, sur les animaux. Le bien être des animaux utilisés dans la recherche doit être respecté.
22. La conception et la conduite de toutes les recherches impliquant des êtres humains doivent être clairement décrites et justifiées dans un protocole de recherche.

Ce protocole devrait contenir une déclaration sur les enjeux éthiques en question et indiquer comment les principes de la présente Déclaration ont été pris en considération. Le protocole devrait inclure des informations concernant le financement, les promoteurs, les affiliations institutionnelles, les conflits d'intérêts potentiels, les incitations pour les personnes impliquées dans la recherche et des informations concernant les mesures prévues pour soigner et/ou dédommager celles ayant subi un préjudice en raison de leur participation à la recherche.

Dans les essais cliniques, le protocole doit également mentionner les dispositions appropriées prévues pour l'accès à l'intervention testée après l'essai clinique.

6 Comités d'éthique de la recherche

23. Le protocole de recherche doit être soumis au comité d'éthique de la recherche concerné pour évaluation, commentaires, conseils et approbation avant que la recherche ne commence. Ce comité doit être transparent dans son fonctionnement, doit être indépendant du chercheur, du promoteur et de toute autre influence indue et doit être dûment qualifié. Il doit prendre en considération les lois et réglementations du ou des pays où se déroule la recherche, ainsi que les normes et standards internationaux, mais ceux-ci ne doivent pas permettre de restreindre ou exclure l'une des protections garanties par la présente Déclaration aux personnes impliquées dans la recherche.

Le comité doit avoir un droit de suivi sur les recherches en cours. Le chercheur doit fournir au comité des informations sur le suivi, notamment concernant tout évènement indésirable grave. Aucune modification ne peut être apportée au protocole sans évaluation et approbation par le comité. A la fin de la recherche, les chercheurs doivent soumettre au comité un rapport final contenant un résumé des découvertes et des conclusions de celle-ci.

7 Vie privée et confidentialité

24. Toutes les précautions doivent être prises pour protéger la vie privée et la confidentialité des informations personnelles concernant les personnes impliquées dans la recherche.

8 Consentement éclairé

25. La participation de personnes capables de donner un consentement éclairé à une recherche médicale doit être un acte volontaire. Bien qu'il puisse être opportun de consulter les membres de la famille ou les responsables de la communauté, aucune personne capable de donner un consentement éclairé ne peut être impliquée dans une recherche sans avoir donné son consentement libre et éclairé.

26. Dans la recherche médicale impliquant des personnes capables de donner un consentement éclairé, toute personne pouvant potentiellement être impliquée doit être correctement informée des objectifs, des méthodes, des sources de financement, de tout éventuel conflit d'intérêts, des affiliations institutionnelles du chercheur, des bénéfices escomptés et des risques potentiels de la recherche, des désagréments qu'elle peut engendrer, des mesures qui seront prises après à l'essai clinique et de tout autre aspect pertinent de la recherche. La personne pouvant potentiellement être impliquée dans la recherche doit être informé de son droit de refuser d'y participer ou de s'en retirer à tout moment sans mesure de rétorsion. Une attention particulière devrait être accordée aux besoins d'informations spécifiques de chaque personne pouvant potentiellement être impliquée dans la recherche ainsi qu'aux méthodes adoptées pour fournir les informations. Lorsque le médecin ou une autre personne qualifiée en la matière a la certitude que la personne concernée a compris les informations, il doit alors solliciter son consentement libre et éclairé, de préférence par écrit. Si le consentement ne peut pas être donné par écrit, le consentement non écrit doit être formellement documenté en présence d'un témoin.

Toutes les personnes impliquées dans des recherches médicales devraient avoir le choix d'être informées des conclusions générales et des résultats de celles-ci.

27. Lorsqu'il sollicite le consentement éclairé d'une personne pour sa participation à une recherche, le médecin doit être particulièrement attentif lorsque cette dernière est dans une relation de dépendance avec lui ou pourrait donner son consentement sous la contrainte. Dans ce cas, le consentement éclairé doit être sollicité par une personne qualifiée en la matière et complètement indépendante de cette relation.

28. Lorsque la recherche implique une personne incapable de donner un consentement éclairé, le médecin doit solliciter le consentement éclairé de son représentant légal. Les personnes incapables ne doivent pas être incluses dans une recherche qui n'a aucune chance de leur être bénéfique sauf si celle-ci vise à améliorer la santé du groupe qu'elles représentent, qu'elle ne peut pas être réalisée avec des personnes capables de donner un consentement éclairé et qu'elle ne comporte que des risques et des inconvénients minimes.

29. Lorsqu'une personne considérée comme incapable de donner un consentement éclairé est en mesure de donner son assentiment concernant sa participation à la recherche, le médecin doit solliciter cet assentiment en complément du consentement de son représentant légal. Le refus de la personne pouvant potentiellement être impliquée dans la recherche devrait être respecté.

30. La recherche impliquant des personnes physiquement ou mentalement incapables de donner leur consentement, par exemple des patients inconscients, peut être menée uniquement si l'état physique ou

mental empêchant de donner un consentement éclairé est une caractéristique nécessaire du groupe sur lequel porte cette recherche.

Dans de telles circonstances, le médecin doit solliciter le consentement éclairé du représentant légal. En l'absence d'un représentant légal et si la recherche ne peut pas être retardée, celle-ci peut être lancée sans le consentement éclairé. Dans ce cas, le protocole de recherche doit mentionner les raisons spécifiques d'impliquer des personnes dont l'état les rend incapables de donner leur consentement éclairé et la recherche doit être approuvée par le comité d'éthique de la recherche concerné. Le consentement pour maintenir la personne concernée dans la recherche doit, dès que possible, être obtenu de la personne elle-même ou de son représentant légal.

31. Le médecin doit fournir des informations complètes au patient sur la nature des soins liés à la recherche. Le refus d'un patient de participer à une recherche ou sa décision de s'en retirer ne doit jamais nuire à la relation patient-médecin.

32. Pour la recherche médicale utilisant des tissus ou des données d'origine humaine, telles que les recherches sur tissus et données contenues dans les biobanques ou des dépôts similaires, les médecins doivent solliciter le consentement éclairé pour leur analyse, stockage et/ou réutilisation. Il peut se présenter des situations exeptionnelles où il est impraticable, voire impossible d'obtenir le consentement. Dans de telles situations, la recherche peut être entreprise uniquement après évaluation et approbation du comité d'éthique de la recherche concerné.

9 Utilisation de placebo

33. Les bénéfices, les risques, les inconvénients, ainsi que l'efficacité d'une nouvelle intervention doivent être testés et comparés à ceux des meilleures interventions avérées, sauf dans les circonstances suivantes :

lorsqu'il n'existe pas d'intervention avérée, l'utilisation de placebo, ou la non intervention, est acceptable ; ou

lorsque pour des raisons de méthodologie incontournables et scientifiquement fondées l'utilisation de toute intervention moins efficace que la meilleure éprouvée, l'utilisation d'un placebo, ou la non intervention, est nécessaire afin de déterminer l'efficacité ou la sécurité d'une intervention,

et lorsque les patients recevant une intervention moins efficace que la meilleure éprouvée, un placebo, ou une non intervention, ne courent pas de risques supplémentaires de préjudices graves ou irréversibles du fait de n'avoir pas reçu la meilleure intervention éprouvée.

Le plus grand soin doit être apporté afin d'éviter tout abus de cette option

10 Conditions de l'accès à l'intervention testée après l'essai clinique

34. En prévision d'un essai clinique, les promoteurs, les chercheurs et les gouvernements des pays d'accueil devraient prévoir des dispositions pour que tous les participants qui ont encore besoin d'une intervention identifiée comme bénéfique dans l'essai puissent y accéder après celui-ci. Cette information doit également être communiquée aux participants au cours du processus de consentement éclairé.

11 Enregistrement des recherches, publication et dissémination des résultats

35. Toute recherche impliquant des êtres humains doit être enregistrée dans une banque de données accessible au public avant que ne soit recrutée la première personne impliquée dans la recherche.

36. Les chercheurs, auteurs, promoteurs, rédacteurs et éditeurs ont tous des obligations éthiques concernant la publication et la dissémination des résultats de la recherche. Les chercheurs ont le devoir de mettre à la disposition du public les résultats de leurs recherches impliquant des êtres humains. Toutes les parties ont la responsabilité de fournir des rapports complets et précis. Ils devraient se conformer aux directives acceptées en matière d'éthique pour la rédaction de rapports. Les résultats aussi bien négatifs et non concluants que positifs doivent être publiés ou rendus publics par un autre moyen. La publication doit mentionner les sources de financement, les affiliations institutionnelles et les conflits d'intérêts. Les rapports de recherche non-conformes aux principes de la présente Déclaration ne devraient pas être acceptés pour publication.

12 Interventions non avérées dans la pratique clinique

37. Dans le cadre du traitement d'un patient, faute d'interventions avérées ou faute d'efficacité de ces interventions, le médecin, après avoir sollicité les conseils d'experts et avec le consentement éclairé du patient ou de son représentant légal, peut recourir à une intervention non avérée si, selon son appréciation professionnelle, elle offre une chance de sauver la vie, rétablir la santé ou alléger les souffrances du patient. Cette intervention devrait par la suite faire l'objet d'une recherche pour en évaluer la sécurité et l'efficacité. Dans tous les cas, les nouvelles informations doivent être enregistrées et, le cas échéant, rendues publiques.
